# Probing ionic conductivity and electric field screening in perovskite solar cells: a novel exploration through ion drift currents†

**DOI:** 10.1039/d4ee02494j

**Published:** 2024-12-17

**Authors:** Matthias Diethelm, Tino Lukas, Joel Smith, Akash Dasgupta, Pietro Caprioglio, Moritz Futscher, Roland Hany, Henry J. Snaith

**Affiliations:** a Department of Physics, University of Oxford, Clarendon Laboratory Oxford OX1 3PU UK mat.diethelm@gmail.com henry.snaith@physics.ox.ac.uk; b Empa, Swiss Federal Laboratories for Materials Science and Technology, Laboratory for Thin Films and Photovoltaics CH-8600 Dübendorf Switzerland; c Empa, Swiss Federal Laboratories for Materials Science and Technology, Laboratory for Functional Polymers CH-8600 Dübendorf Switzerland

## Abstract

It is widely accepted that mobile ions are responsible for the slow electronic responses observed in metal halide perovskite-based optoelectronic devices, and strongly influence long-term operational stability. Electrical characterisation methods mostly observe complex indirect effects of ions on bulk/interface recombination, struggle to quantify the ion density and mobility, and are typically not able to fully quantify the influence of the ions upon the bulk and interfacial electric fields. We analyse the bias-assisted charge extraction (BACE) method for the case of a screened bulk electric field, and introduce a new characterisation method based on BACE, termed ion drift BACE. We reveal that the initial current density and current decay dynamics depend on the ion conductivity, which is the product of ion density and mobility. This means that for an unknown high ion density, typical in perovskite solar absorber layers, the mobility cannot be directly obtained from BACE measurements. We derive an analytical model to illustrate the relation between current density, conductivity and bulk field screening, supported by drift–diffusion simulations. By measuring the ion density independently with impedance spectroscopy, we show how the ion mobility can be derived from the BACE ion conductivity. We highlight important differences between the low- and high-ion density cases, which reveal whether the bulk electric field is fully screened or not. Our work clarifies the complex ion-related processes occurring within perovskite solar cells and gives new insight into the operational principles of halide perovskite devices as mixed ionic–electronic conductors.

## Abstract 2

This study introduces a new method to analyse mobile ion-related processes in metal halide perovskite devices, which reveals how ion conductivity determines electric field screening and provides new insights into the mixed ionic–electronic behaviour.

## Graphical abstract

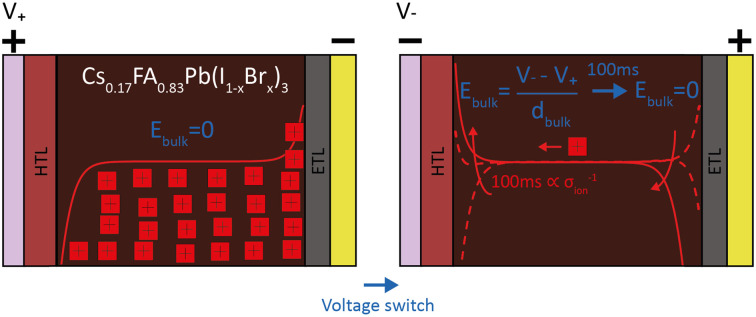

**Broader context**

In recent years, metal halide perovskite-based optoelectronic devices have attracted significant attention due to their potential for high efficiency and low-cost production. It has been recognised that mobile ions are critical material constituents that strongly influence the device response and long-term operational stability. Indeed, the latter can be considered as a significant obstacle to the development of a competitive photovoltaic technology, which is still dominated by stable silicon solar cells. Electrical characterisation techniques have encountered difficulties in directly quantifying ion density and mobility in perovskite materials, as well as in understanding the impact of ions on bulk and interfacial electric fields. This study presents a novel approach based on bias-assisted charge extraction (BACE) to address these challenges. The results show that the time scale of ion-related dynamics is predominantly determined by ion conductivity, defined as the product of ion density and mobility, rather than by ion mobility alone. The method employed allows the extraction of this conductivity and the determination of the extent and details of bulk electric field screening, especially at high mobile ion densities. The combination of impedance spectroscopy measurements with our approach provides a comprehensive understanding and quantification of ion dynamics in perovskite solar cells.

## Introduction

During the meteoric rise in efficiency of metal halide perovskite-based optoelectronic devices to over 26% power conversion efficiency for single-junction solar cells and over 30% external quantum efficiency for light-emitting devices (LEDs), slow transient effects during device operation became apparent.^1,2^ After charge trapping or ferroelectricity were discussed as possible origins,^3^ the presence of mobile ionic species is agreed to be a critical factor to explain, *e.g.*, hysteresis and losses in solar cell performance, or altered electroluminescence in LEDs.^4,5^ The issue appears to be resolved for many high-efficiency solar cells, which show negligible hysteretic behaviour in their current–voltage curves under standard scanning conditions.^6,7^ However, even if hysteresis is not observed, there can still be considerable ionic motion,^8^ or the timescale for ionic motion is too fast or too slow to be revealed at a given scan speed. Furthermore, during aging, the impact of ions is likely to be enhanced through an increase in the ion density^9,10^ and thus increased perturbation of the electric field distribution throughout the device,^10^ as well as a higher trap density.^11,12^

Given the importance and impact of mobile ions on perovskite device performance, it is necessary to have available electrical characterisation techniques that provide a clear picture of the role of ions during device operation, which begins with accurately measuring ion density and mobility values.^9,13,14^ A problem is that several experimental methods trace indirect effects of ions on bulk/interface recombination or dark injection currents, from which it is difficult to directly quantify the ion density and mobility. For example, it is accepted that large changes in the imaginary part of the admittance (
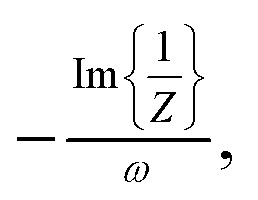
 in the following referred to as capacitance) measured by impedance spectroscopy at low frequencies are a result of space charge and thus electric field variation due to ionic movement along the small applied sinusoidal voltage.^14–17^ A large increase in capacitance is linked to ion-induced changes in interface recombination,^14,16,17^ while a negative capacitance is associated with injection modulation.^14,16^ Ion-related parameters are extracted by equivalent circuit fitting,^18,19^ and while fitting across different material systems and devices is viable beyond solar cells,^20^ assigning a physical meaning to fitting elements is challenging.^21^ Drift–diffusion simulation is able to physically quantify the interactions properly, but cannot be used as a routine method due to the vastness of model parameters and the necessity to fit several different characterisation methods to achieve a consistent picture.^22^ Furthermore, some likely relevant ion-related processes are not well understood and are therefore typically not introduced into the simulation models, specifically the interaction between transport layers (TLs) and depletion regions,^23^ or effects arising when ions penetrate TLs.^24–26^

To isolate the indirect effects of ions on electronic currents, measurements should be carried out in a region where electronic currents are negligible and do not influence the measurement. This is generally the case in the dark and at voltages below injection levels. In bias-assisted charge extraction (BACE), the voltage is suddenly switched from a preconditioning to an extraction voltage, leading to a redistribution of the ions. This causes a change of the internal electric field, which results in a current in the external circuit that is equal to the ion current.^9,10,27–29^ To date, BACE analysis has only been feasible for an ion density that is lower than the limit necessary to screen the electric field in the bulk.^9,10^ Screening of the bulk electric field as a result of ions piling up at the interfaces has been shown in combined electrical and computational characterisation studies^14,27^ and also with direct measurements.^30,31^ In this high ion density regime, a modified Mott–Schottky analysis is arguably the most reliable method for measuring ion density.^9^ Thereby, capacitance levels that depend on the ion density are measured by impedance spectroscopy analysis at low frequency in the dark.^9,13,32^

Nevertheless, our findings show that focusing solely on ion density is insufficient to fully comprehend the dynamic device operation. Specifically, we explain that the influence of mobile ions is not governed only by the ion density or the ion mobility, but rather by their product, the ion conductivity. We derive an analytical model to illustrate the relation between ion current density, ion conductivity and bulk electric field screening. With the ion density independently measured from impedance spectroscopy, the ion mobility can then be obtained from the BACE conductivity. These findings result in a new characterisation method based on BACE, which focuses on the previously unexplored drift current density, carefully analysed by experimental electric field modulations and drift–diffusion simulations. The analysis reveals – in addition to ionic conductivity – information specifically about the bulk electric field screening, which is an important aspect to understanding the device behaviour.^30^

## Results

**Fig. 1:**
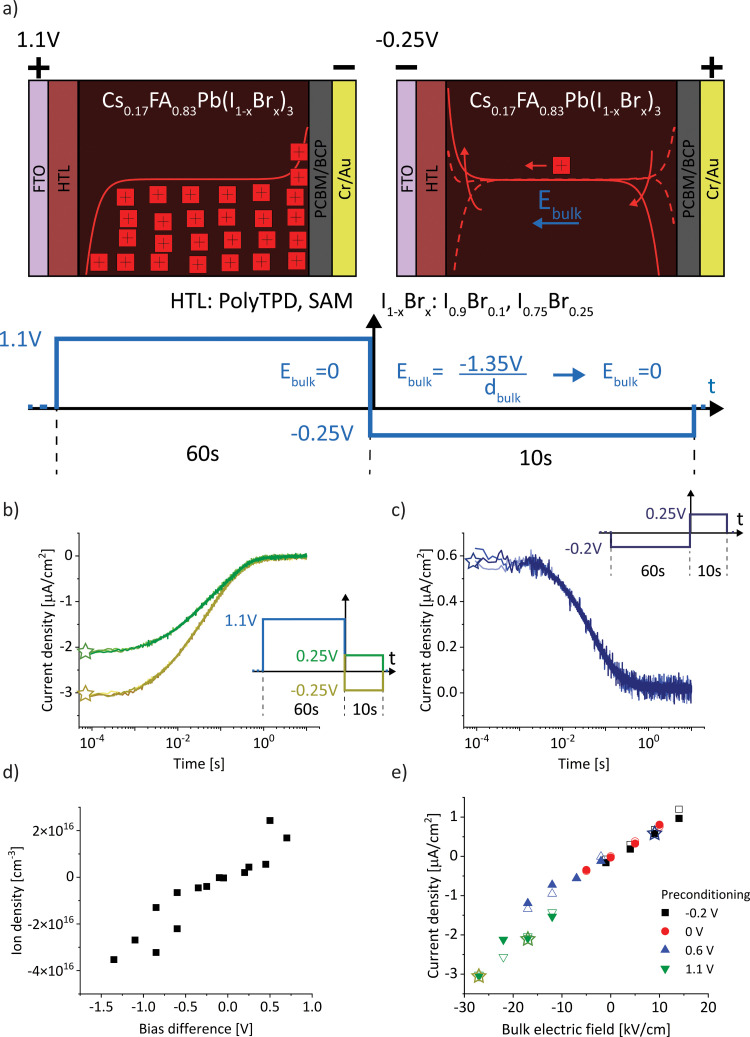
(a) Schematic of the bias-assisted charge extraction (BACE) measurement. A device is preconditioned for 60 s at 1.1 V (in the dark), followed by measurement of the current after a voltage switch to −0.25 V. During preconditioning and after the voltage switch, the ions redistribute until drift and diffusion match and the electric field is zero everywhere. Arrows indicate the ion movement direction. (b) Measured current after a bias switch from 1.1 V to −0.25 V (yellow) or to 0.25 V (green). (c) Measured current after preconditioning at −0.2 V, followed by a switch to 0.25 V. Each measurement is repeated several times to demonstrate the reproducibility (overlapping lines with slightly different colour in (b) and (c)). (d) Ion density *versus* bias difference (bias after switch minus preconditioning bias). (e) Compilation of BACE measurement and drift–diffusion simulation results (open symbols). The current density levels at early times (<10^−3^ s) for 16 measurements and simulations for each combination of preconditioning (−0.2, 0, 0.6 and 1.1 V) and measurement voltage (−0.25, 0, 0.25 and 0.5 V) are plotted against the bulk electric field (bias difference divided by the bulk thickness). Data points from (b) and (c) are marked with stars, the full set of transient measurements is shown in the Fig. S3 (ESI†).

For a sufficiently high ion density that is able to fully screen the applied electric field, a change in electric bias leads to a redistribution of ionic charge close to the interfaces while the bulk ion density remains almost constant, as indicated in Fig. 1a. The schematic describes a bias-assisted charge extraction (BACE) measurement with a 60 s preconditioning bias at 1.1 V, followed by a switch to −0.25 V. This preconditioning bias is close to the open-circuit voltage (*V*_OC_ ≈ 1.15 V) of the device, which results in a homogeneous ion density throughout the active layer and only few excess ions accumulating at the interfaces. The voltage change results in an instantaneous bulk electric field of ≈−1.35 V/*d*_bulk_, which is the voltage difference between the applied bias and the preconditioning bias, divided by the perovskite bulk thickness *d*_bulk_. This bulk electric field is subsequently screened by the displacement of ionic charge. We assume positively charged halide vacancies as mobile pseudo-cations that dominate the ionic response. This assumption is supported by temperature-dependent measurements shown in Fig. S1 (ESI†), where an activation energy of 0.6 eV is extracted, a value typically associated with the migration of iodide vacancies.^32–34^ The experimental current transient of this BACE measurement is shown in Fig. 1b (yellow), performed on a perovskite solar cell with the structure FTO/polyTPD:F4-TCNQ/Al_2_O_3_/Cs_0.17_FA_0.83_Pb(I_0.9_Br_0.1_)_3_:[BMP]^+^[BF_4_]^−^/PCBM/BCP/Cr/Au; chemical abbreviations are detailed in the Experimental section. The cell exhibits a power conversion efficiency of 18.7% and a maximum power point tracking efficiency of 18.0%, the current–voltage (*J*–*V*) hysteresis measurement and external quantum efficiency (EQE) are shown in the Fig. S2 (ESI†). Processes at timescales below 10^−4^ s were not measured here; these include the charging current due to the voltage switch, as well as the extraction current due to electrons and holes present inside the device.^9,27^

The measured current in the external circuit is interpreted as the response to the drift of ionic charges in the active layer. The current decays over time while the ions rearrange and are blocked at the interface.^9,27,29^ The timescale of the current decay is proportional to the ion mobility (or the diffusion coefficient).^27,29^ Integration of the experimental current transient yields the total ionic charge (*Q*_ion_) per unit area (*A*) that drifts within the active layer to the interface,1
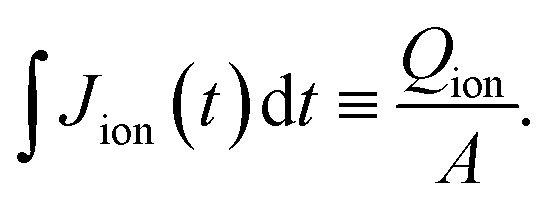
From this, the ion density *n*_ion_ [cm^−3^] can be calculated by dividing by the active layer thickness, 
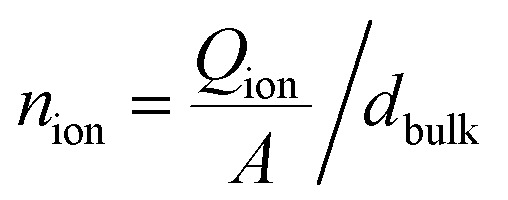
. This ion density represents a lower limit, because the integrated mobile ionic charge corresponds only to a fraction of the total ion density in the active layer.^9^

If after preconditioning the bias is switched to 0.25 V (Fig. 1b, green), the initial current density is lowered to ≈2 μA cm^−2^. The initial current levels at ≈10^−4^ s in Fig. 1b are proportional to the difference between the preconditioning and extraction voltage. We measured the same current transients from Fig. 1b when the BACE experiment was repeated three times with a rest period of 100 s at open-circuit in between. This ensures that the measurement does not induce device degradation and that the displacement of ionic species in the active layer is fully reversible (Supplementary Note SN1, ESI†).^35–37^ Other sources for the observed current transient can be discarded. These include the dark recombination current *J*_0_ and the release of trapped charges, which are too small^38^ and occur at different timescales,^39^ respectively (Supplementary Note SN2, ESI†).

Changing the polarity between the preconditioning and extraction voltage inverts the trend in current, as expected (Fig. 1c). Also in this case, the initial current was proportional to the applied voltage difference. Integration of each current transient (Fig. S3, ESI†) and plotting the calculated ion density *versus* the bias difference reveals a linear trend (Fig. 1d). This supports our assumption that the ion density is high enough to fully screen the applied electric field. The linear trend describes the definition of a capacitance *Q* = *CU*. For every BACE measurement, the applied bias difference Δ*U* is screened by the charge *Q* at the interface with capacitance *C*. If the ion density were small, field screening would not be complete and the bulk would be depleted of ions for preconditioning voltages much lower than *V*_OC_.^27^ In this case, a steady ion density *versus* bias difference trend would not be expected.^9^

Fig. 1e summarizes initial current densities for the combination of four preconditioning and four extraction voltages, plotted against the calculated bulk electric field *E*_bulk_. The linear dependence reveals two important conclusions. First, by definition, this linear dependence of ion current density (*J*_ion_) on the bulk electric field is the ion conductivity2
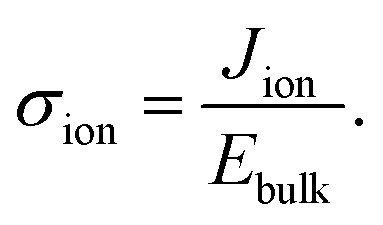
Second, the linear trend of the BACE measurement currents indicates that the device operates under the same process across all voltage levels, which we identified as bulk electric field screening due to ions. We note a minor deviation for all measurements at a preconditioning of 1.1 V (discussed in Fig. 5), but the overall trend and slope, which represents ion conductivity, remain largely similar.

**Fig. 2:**
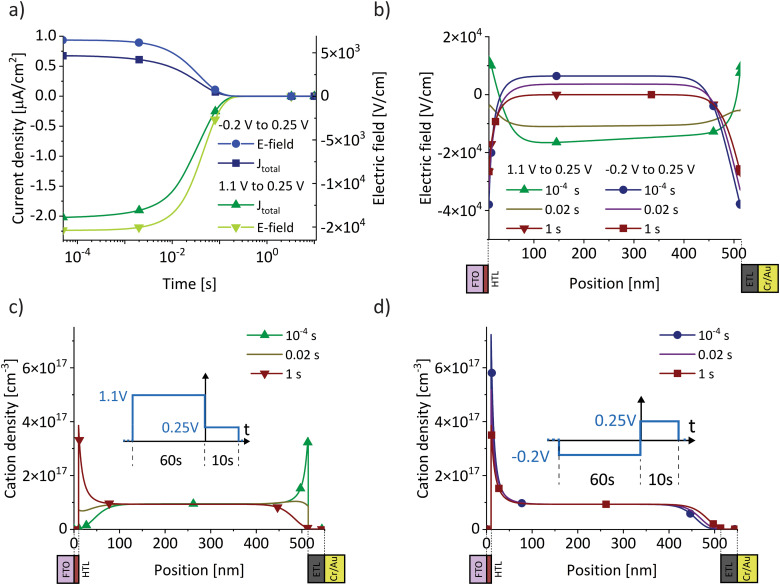
Drift–diffusion simulations with the same protocol as used for the BACE current density measurements in Fig. 1b (green) and Fig. 1c. (a) Current density together with the electric field in the centre of the bulk. Both BACE simulations use the same extraction voltage of 0.25 V and therefore show the same steady-state situation at 1 s for the electric field profiles in (b) and the cation profiles in (c) and (d). Additionally, the situation is shown before the current level decreases at 10^−4^ s, and during the decrease at 0.02 s.

We analyse the experimental findings with the help of drift–diffusion simulation (Driftfusion^40,41^). The parameters used in the simulation, along with the rationale for their selection, are detailed in the Supplementary Note SN3 (ESI†). Simulation results for the two BACE measurements from Fig. 1b (green) and Fig. 1c are shown in Fig. 2a. Since both measurements switch to 0.25 V, but from a higher and lower preconditioning voltage, the induced bulk electric field is negative for the 1.1 V preconditioning voltage, but positive for the −0.25 V preconditioning voltage. In both cases, the simulated current density in the bulk is due to an ionic drift current, *J*_ion_ = *σ*_ion_ × *E*_bulk_. The simulation accurately replicates the experimental initial current levels (Fig. 1e). Hole and electron currents are negligible, ranging from 10^−6^ μA cm^−2^ up to 10^−1^ μA cm^−2^ for the highest applied bias of 0.5 V (Fig. S4, ESI†). Furthermore, the ion diffusion current in the bulk is zero due to the absence of an ion density gradient in the bulk. This is not the case in the interface region, where strong ion density gradients and electric fields exist that lead to opposing diffusion and drift currents. However, since the total current density must remain constant across the device, we can disregard the complex interface region and concentrate on the bulk currents to understand the measured current.

The decrease in bulk current density is attributed to the decline of the bulk electric field, as depicted in Fig. 2a. At any given time, the bulk electric field remains relatively constant over large parts of the bulk and only varies at the interface (Fig. 2b). The bulk electric field at early times is due to the applied bias difference, hence dividing the bias difference by the bulk thickness provides a good approximation for the initial bulk electric field (
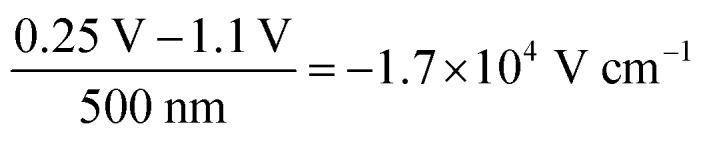
 and 
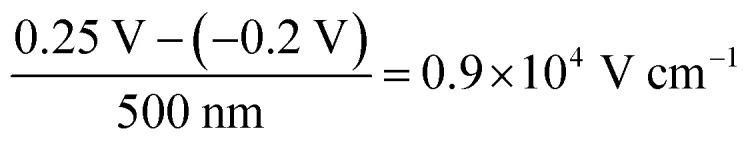
). The accumulation and depletion of cations in the interface region, as illustrated in Fig. 2c and d, account for the electric field variation near the interface over time. It is important to note that the cations in the bulk are compensated by an immobile anionic counterpart of the same density. Therefore, the cations do not contribute to the electric field in the bulk region.

Once the bias is switched and the bulk electric field is present, all cations move, leading to a constant ion current density level. This continues until the ions accumulate at or deplete from the interface region and the resulting electric field screens the bulk field. In Fig. 2b, this is illustrated by the fact that the bulk and interface electric fields move in opposite directions over time. The cations do not move across the entire device, rather each cation only moves a small fraction of the bulk thickness. The bulk ion density remains unchanged because only a fraction of the ions close to each interface are necessary to screen the bulk electric field. This fraction depends on the bulk ion density and the ionic charge necessary to screen the electric field.

Introducing a mobile anionic species into the model does not alter the simulated behaviour qualitatively. Also, it only has a quantitative impact if the addition of anions increases the total ion conductivity of the system, as demonstrated in Fig. S5a (ESI†). For instance, this is not true when the density of anions is equal to that of cations but with significantly lower mobility.

Understanding if ions can penetrate the transport layers is vital for interpreting their influence on the current–voltage measurements of a solar cell during operation.^26^ However, the ability of cations to infiltrate the TLs has a very small effect on the overall electric field screening and thus on results from BACE simulations (Fig. S5c, ESI†). Therefore, our conclusions are not influenced by whether ions penetrate the TLs or not.

## Discussion

An important conclusion from the simulation is that the individual choices for the bulk ion density *n*_ion_ and the ion mobility *μ*_ion_ only result in relatively small variations of the initial current level and decline timescale as long as the ion conductivity,

3 *σ*_ion_ = *q* × *n*_ion_ × *μ*_ion_

remains constant (Fig. S6, ESI,†*q*: charge). However, the ion density in the simulation has to be above the limit for field screening and should be above the experimental integrated charge density (Fig. 1d); here we chose 1 × 10^17^ cm^−3^, which agrees with reported values between 10^16^ cm^−3^ and 10^17^ cm^−3^.^9^ In the model, an ion mobility of 10^−8^ cm^2^ V^−1^ s^−1^ matched the experimental data well, which is in the range of reported values.^27,29^ While the relation between the mobile ion-induced current level and conductivity is simple (eqn (2)), the relation between the decaying current dynamics and the ion conductivity is intricate and cannot be readily deduced from the simulation results. In the following, an analytical model is derived which shows the key elements to understand this relation.

The bias switch creates an electric field in the bulk material, which sets ions in motion and results in an ion drift current *J*_ion_. This current leads to accumulation of ionic charge at the interface. The speed at which the voltage across the interface *U*_*C*_ion__ builds up depends on how quickly this ionic charge accumulates, which determines how fast the electric field in the bulk material is screened. The charge accumulation and voltage drop at the interface are related through the ionic interface capacitance per unit area, *C*_ion_. This relationship can be expressed as:4
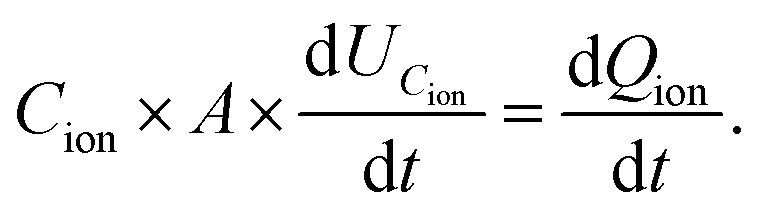
The voltage drop at the interface and the bulk electric field are related by

5 *U*_applied_ − *U*_*C*_ion__ = *U*_bulk_ = *E*_bulk_ × *d*_bulk_,

where we neglect changes in the potential drop across the TLs over time, and also across the perovskite/TL interfaces.^14,21^ Substituting eqn (5) into eqn (4) and expressing 
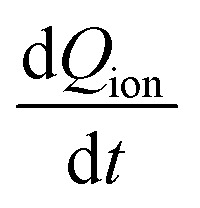
 as 
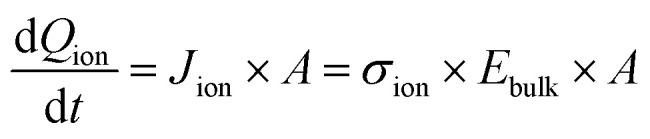
 leads to6
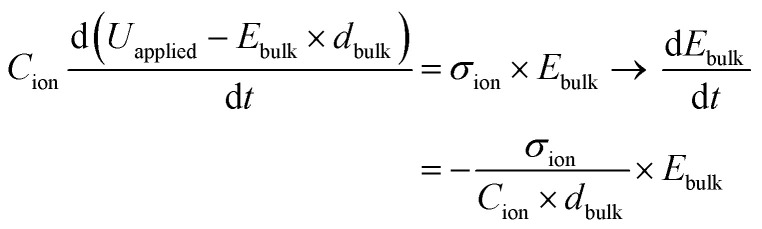
using 
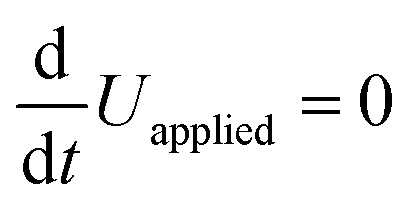
. Eqn (6) demonstrates that a higher initial bulk electric field *E*_bulk_ results in a faster decaying field 
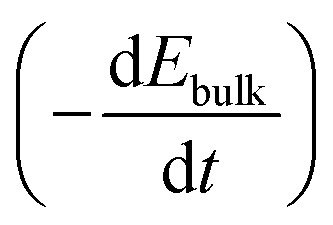
, as a result of a higher ion current. Solving this differential equation leads to an exponentially decreasing 
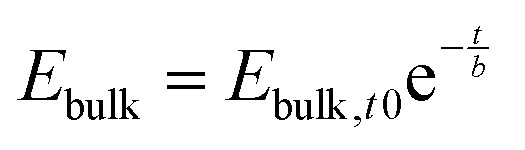
 with the time constant7
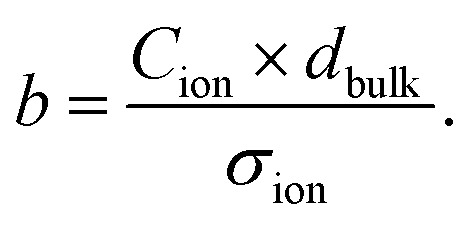
We can now express the measured BACE current as 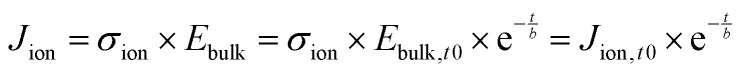 and discuss individually the current density level *J*_ion,*t*0_ = *σ*_ion_ × *E*_bulk,*t*0_ and the timescale of its decrease with the time constant 
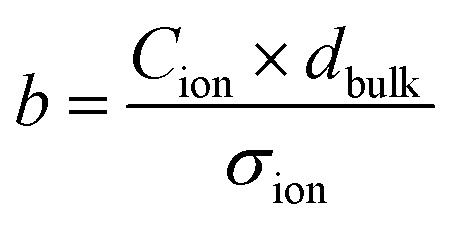
. Note, by calculating a specific resistance to ion motion across the perovskite layer 
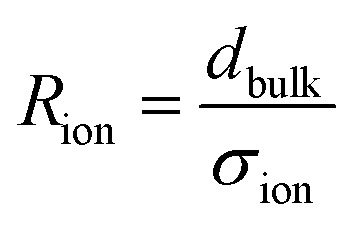
, the time constant becomes *b* = *C*_ion_ × *R*_ion_, which is typically used in equivalent circuit model elements to describe the ionic circuit branch of the impedance.^14^ Also, the term *C*_ion_ × *d*_bulk_ can be reduced to an equivalent low frequency bulk permittivity *ε*_0_*ε*_s_,^14^ which should not be confused with the high-frequency permittivity *ε*_0_*ε*_r_ = *C*_g_ × *d*_bulk_. This allows the definition of the time constant to be expressed as 
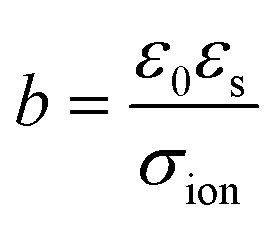
, as similarly used in previous work.^42^

**Fig. 3:**
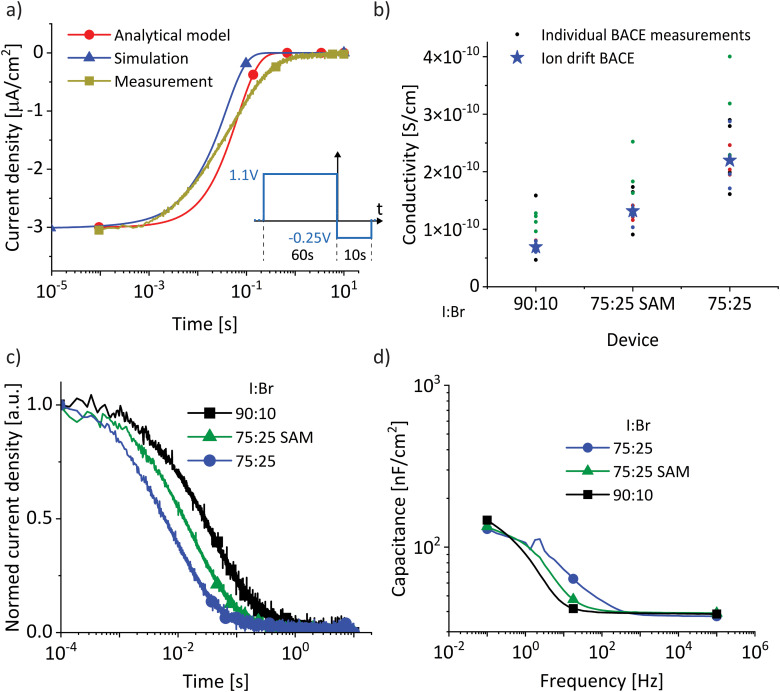
(a) Comparison of the experimental BACE current transient (from Fig. 1b, yellow curve) with simulated transients using drift–diffusion simulation (blue) and the analytical model (red). (b) Ionic conductivity calculated from the individual initial BACE current density levels. The stars represent calculated values from the slope of BACE measurements in Fig. 1e with preconditioning biases below 1.1 V. The device structures are shown in Fig. 1a where the I : Br ratio and the hole transport layer is varied. (c) BACE measurements at 0 V after a preconditioning bias of 0.6 V for different devices. (d) Capacitance measurement in the dark and at 0 V.

We compare the analytical model to the simulation and the experimental data in Fig. 3a. The time constant is 
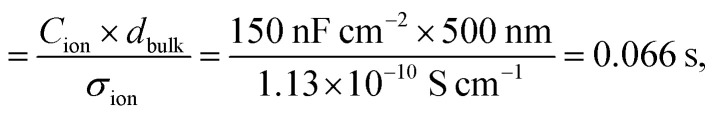
 calculated with the measured interface capacitance *C*_ion_ = 150 nF cm^−2^ (impedance spectroscopy at 0 V, dark and low frequency, Fig. 3d), the thickness of the bulk material (500 nm, scanning electron microscopy^43^), and the calculated ion conductivity of 1.13 × 10^−10^ S cm^−1^ (BACE measurement, voltage switch from 1.1 V to −0.25 V). With this, the current dynamics calculated with the analytical model agrees very well with the experimental and simulated trends (Fig. 3a).

With the intention to slightly alter the ionic conductivity, we repeated the whole study using a device with the same structure but a different I : Br ratio of 75 : 25 (Fig. S7, ESI†). Furthermore, to study the influence of different transport layers, we measured a I : Br = 75 : 25 device but replaced polyTPD with a self-assembled monolayer (SAM) of Me-4PACz (see Experimental section).^44,45^ For all devices we observed the same trends and draw the same conclusions on the relation between ion current density, bulk field screening and ion conductivity (Fig. S8, ESI†). Decreasing the iodide to bromide ratio has been shown to decrease the activation energy for ion migration, which in turn increases the ionic conductivity.^33,46^ In Fig. 3b, we observe this expected trend in the calculated conductivity from the different BACE current density levels. Comparing devices with the same I : Br ratio, the SAM layer resulted in a slightly lower conductivity. This can possibly be ascribed to differences in crystallization of the perovskite on the TLs, or due to subtle experimental variations (see Experimental section).

The observed conductivity trends are confirmed by two further observations. First, Fig. 3c shows BACE measurements at 0 V after a preconditioning bias of 0.6 V for the three devices, normalized to focus on the decay time. The I : Br = 75 : 25 device with the highest conductivity shows the fastest timescale in the BACE current decrease, as predicted by eqn (7). Second, Fig. 3d shows the calculated capacitance from impedance spectroscopy in the dark and at 0 V for the three devices. The capacitance level at frequencies too high for ionic movement above 10^3^ Hz is dominated by the geometric capacitance *C*_g_, which describes the electronic charging of the plate capacitor-like device structure. The level at the lowest frequencies is related to the ion accumulation layer^14,42^ with the associated interface capacitance *C*_ion_. The frequencies at which the capacitance increases from *C*_g_ to *C*_ion_ are therefore related to electric field-induced (*via* the applied small sinusoidal voltage) movement of ions to the interface and thus ion conductivity. Again, the I : Br = 75 : 25 device with the highest conductivity also shows the highest transition frequency.

**Fig. 4:**
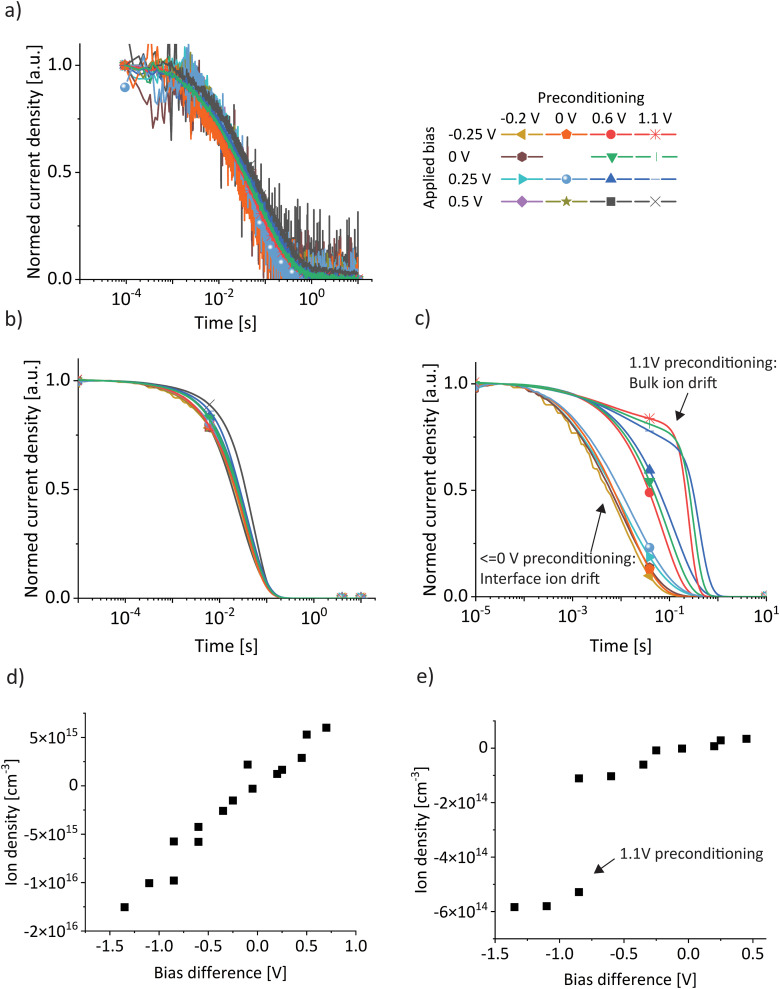
(a) Normalized experimental and (b) simulated BACE measurements in the high ion density regime. (c) BACE drift–diffusion simulations with a 2 orders of magnitude lower ion density (10^15^ cm^−3^). (d) and (e) Ion density resulting from the integrated BACE current density simulations for the high and low ion density regime, respectively.

From the ion conductivity the explicit values for the ion mobility and density can be calculated provided that one of these two parameters is already known. However, it is important to note that the ion mobility cannot be directly calculated from the timescale of the current decrease. According to eqn (6), the decline of the bulk electric field 
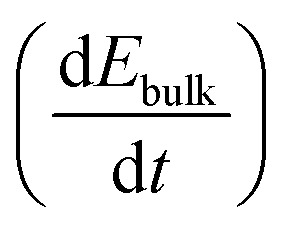
, and consequently the ion current density, linearly depends on the ion conductivity. This means it depends on the product of ion mobility and density, not just on the ion mobility alone. Often, ion mobility is estimated from the timescale of current decrease and an assumed distance the ions travel through the active layer, which leads to the drift velocity. Mobility is then calculated as it is, by definition, the proportional constant of the drift velocity divided by the electric field. This has its validity in the case of a low ion density.^27,29^ However, in our case, the normalized BACE data for both the experiment (Fig. 4a) and the simulation (Fig. 4b) show a constant current decay timescale, although the bulk electric field varies by a factor 30 within the measurement and simulation series. In the high ion density range where the bulk electric field is fully screened, the integrated charge from the BACE measurement only reflects the charge that is necessary to screen the electric field. When the bulk electric field is no longer screened, the integrated charge density should reflect the actual bulk ion density.^9^ This is not the case for our devices, but low ion density pristine devices have been reported.^10^

To obtain ion density and mobility from the experimental ionic conductivity in the high ion density region, using BACE together with transient ion drift (TID) measurement at high frequencies^32^ and/or Mott–Schottky analysis at low frequencies^9^ is a promising combination. Both methods use impedance spectroscopy to extract capacitance levels, which are independent of ion mobility but depend on the ion density.^9,13,32^ Since the TID model arguably is not adapted enough to the high ion densities in perovskite films (Supplementary Note SN4, ESI†), we calculated the ion density *via* the Mott–Schottky analysis using impedance spectroscopy at low frequencies at 0 V and 0.3 V (Fig. S9b, ESI†). Alteration of the applied voltage modifies the ionic distribution in the interface region, which in turn affects the interface capacitance. The Mott–Schottky equation, 
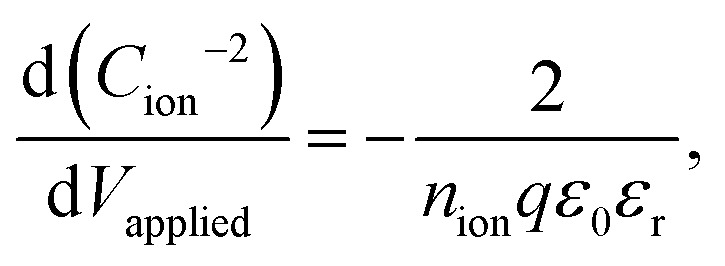
 is then used to calculate the ion density.^9,47,48^ The accuracy of this method for extracting the ion density has been confirmed through a drift–diffusion model study of a perovskite solar cell.^9^Table 1 shows ion parameter calculations for the I : Br = 75 : 25 device with conductivity extracted from the fit of several BACE measurements (Fig. S9a, ESI†).

**Table 1:** Ion conductivity, density and mobility calculation for the I : Br = 75 : 25 device

Conductivity from *J vs. E*_bulk_ BACE	Corrected conductivity	Ion density from Mott–Schottky analysis	Calculated mobility 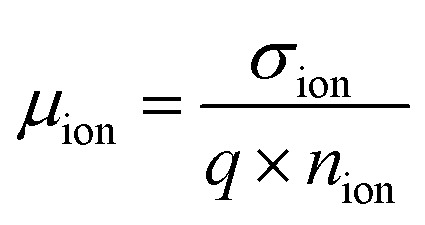
2.2 × 10^−10^ S cm^−1^	4.4 × 10^−10^ S cm^−1^	9.8 × 10^16^ cm^−3^	2.75 × 10^−8^ cm^2^ V^−1^ s^−1^

Note that the simulation study reveals a correction factor of 2 due to two approximations used in the conductivity calculation, as explained in the Supplementary Note SN5 (ESI†) in more detail. First, a displacement current due to the bulk electric field change leads to an underestimation of the actual ion drift current and consequently ion conductivity, such that the measured current has to be corrected by a factor ∼1.4. Second, a voltage drop across transport layers leads to a bulk electric field overestimation and thus ion conductivity underestimation, leading as well to a correction factor of ∼1.4, therefore 1.4 × 1.4 ≈ 2 in total. The extracted parameters for the I : Br = 75 : 25 device (Table 1) are close to the simulation parameters for the I : Br = 90 : 10 device (*n*_ion_ = 9.3 × 10^16^ cm^−3^, *μ*_ion_ = 1 × 10^−8^ cm^2^ V^−1^ s^−1^). This suggests that the measured conductivity difference between these two devices (a factor of ∼3, Fig. 3b) results from a similar difference in the ion mobilities. However, a detailed analysis of the conductivity difference between these two devices would require an extensive Mott–Schottky analysis, which was not carried out and is therefore not discussed further. The aim of the analysis in Table 1 is a proof of concept, the validity of the ion density extraction from Mott–Schottky analysis for perovskite solar cells has been shown elsewhere.^9^

The measured and simulated BACE current declines for the I : Br = 90 : 10 device in Fig. 4a and b show the same timescale, which is also the case for the BACE measurement of the I : Br = 75 : 25 devices (Fig. S10, ESI†). In contrast, Fig. 4c shows a simulation with the same parameters as used for Fig. 4b, but using a two orders of magnitude lower ion density. In this case, the bulk electric field cannot be fully screened over the applied bias range (Fig. S11, ESI†). The normalized BACE current decays no longer show the same timescales, but they are now different and grouped according to the preconditioning condition. When the preconditioning bias is close to *V*_OC_ at 1.1 V, the ions are distributed homogeneously across the device. In this situation, the decay of the current originates from an ion drift current in the bulk which is being depleted of ions. Conversely, the bulk is already depleted during preconditioning at 0 V or at −0.2 V. Therefore, changes are related to ion drift in the interface region, which appear faster. A preconditioning bias of 0.6 V corresponds to the intermediate situation.

A second characteristic difference between the high and low ion density range appears when plotting the integrated ion density *versus* the bias difference. For the high ion density case, both experimental results (Fig. 1d) and simulations (Fig. 4d) reveal a linear trend, which reflects the larger number of ions necessary to screen the increasing bulk electric field.^9^ On the other hand, in the low ion density regime the simulation (Fig. 4e) indicates a sudden increase in integrated ion density. This is because all preconditioning biases below 1.1 V result in an ion-depleted bulk, and as a result, the integrated current density of the BACE measurement primarily originates from ion drift currents in the interface region.

Therefore, integration of the BACE current only yields an estimate of the ion density in the low density range if preconditioning is applied at around *V*_OC_.^9,10^ In this case, the ion distribution is approximately homogeneous and ions drift on average half of the film thickness. Considering this factor of 2, we calculate a bulk ion density of approximately 10^15^ cm^−3^ from the simulation shown in Fig. 4e. This value is consistent with the bulk ion density used as an input parameter for the low ion density simulation. However, this is not the case for the high ion density regime depicted in Fig. 4d, a discrepancy that increases towards higher simulated bulk ion densities.^9^

Estimating the mobility in the low ion density regime from the timescale of the BACE measurement current decrease as described above also has its validity.^27,29^ Here, the simulation shows that it is still possible to extract the ion conductivity in the same way as shown for the high ion density regime (Fig. S12, ESI†). This is because there is still a considerable ion density in the interface region, which leads to a similar screening situation and interface ion drift current when the bias is switched after preconditioning. Extracting the ion conductivity in the low ion density regime could strengthen the extracted ion density and mobility parameters. However, this needs to be confirmed experimentally in further work.

**Fig. 5:**
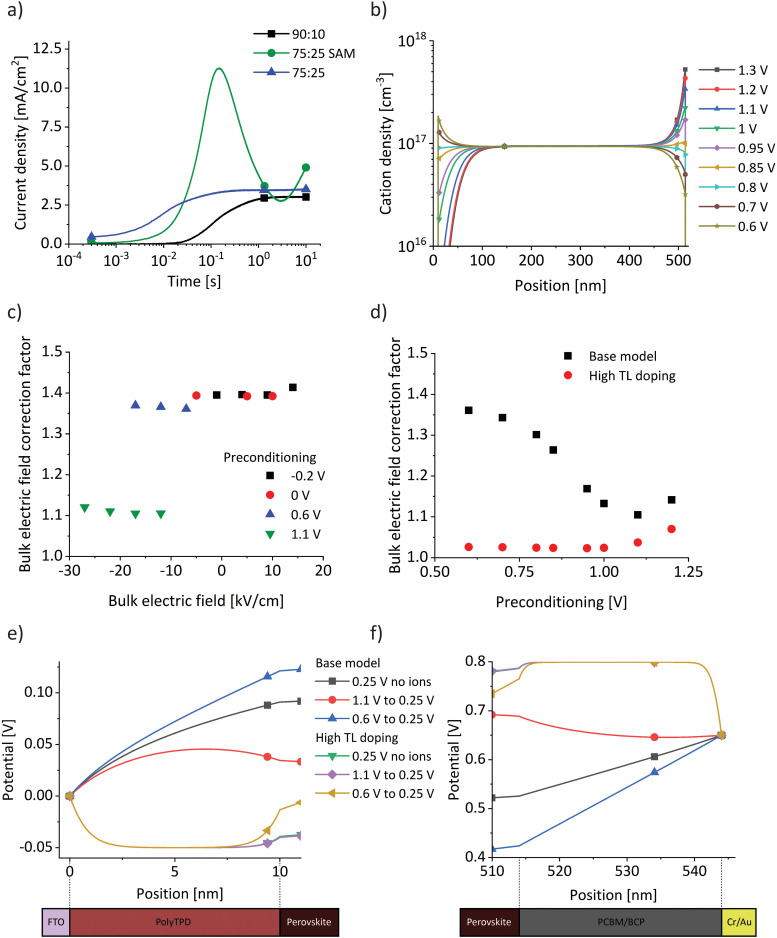
(a) Measured dark injection current increase as a result of a bias switch from 0 V to 1.2–1.3 V (0.1 V above the measured *V*_OC_ of each device) in the dark for the three investigated device structures. (b) Simulated steady-state ion distributions for preconditioning voltages from 1.3 V to 0.6 V of the base model. (c) Deviation of the estimated bulk electric field from the simulated value, 
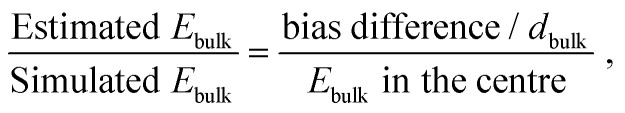
 for the whole set of BACE measurements, labelled as correction factor. (d) Bulk electric field correction factors from the simulation for preconditioning from 1.3 V to 0.6 V and subsequent switching to 0.25 V, for the base model used for all previous simulation results, and a model with high TL doping. (e) and (f) Potential across the transport layers for BACE simulations 10^−4^ s after a switch to 0.25 V from a preconditioning bias of 1.1 V and 0.6 V, for the base model with and without ions. The simulations are repeated for the model with high TL doping. Note that the results for no ions (green) and preconditioning at 1.1 V (purple) overlap.

Our results show that varying the transport layer does not fundamentally alter the BACE measurements. However, changes in TLs significantly influences measured electronic currents during operation, as shown for wide-gap perovskite solar cells when comparing a SAM with other, thicker TLs.^26^ This discrepancy arises because device operation depends on the interplay between the ionic current component measured by BACE and recombination processes, including bulk and interface recombination, as well as dark injection currents. In Fig. 5a the impact of ionic redistribution on measured electronic currents in the dark is observed, when the bias voltage is switched for the three device structures from 0 V to 1.2–1.3 V (0.1 V above the measured *V*_OC_ of each device). Initially, the dark current is very low due to a potential barrier, which is reduced as the ions begin to drift. Such a potential barrier can occur at the perovskite/TL interface due to the strong influence of ionic charge, which alter the interface band alignment.^14^ The difference in the timescale of the initial dark current rise is consistent with the ion conductivity analysis in Fig. 3. Comparing the 90 : 10 device with the 75 : 25 device shows a similar response, the only difference being a faster dark current rise of the 75 : 25 device due to the higher ionic conductivity. The 75 : 25 SAM device, however, exhibits a different behaviour in electronic current in alignment with the discussion above.

An unexplored interaction between ions and TLs emerges when comparing preconditioning at 1.1 V with those at ≤0.6 V (Fig. 1e and Fig. S8, ESI†). For all device architectures a shift towards higher currents is observed for a preconditioning at 1.1 V. In Fig. 1e, the close match between experimental and simulated data indicates that the model also reflects this current offset. The fundamental difference in the simulation when increasing the preconditioning bias from ≤0.6 V to 1.1 V lies in the crossing of the structural built-in potential of 0.9 V in the model, which arises from the energy level alignment of all layers in the absence of ions. We indicate this as a structural built-in potential because, unlike inorganic semiconductor solar cells, our devices do not have a built-in potential, as ions screen the electric field in the bulk. The structural built-in potential determines the preconditioning bias at which ions begin to accumulate at the opposite interface. Fig. 5b illustrates ion distributions for preconditioning voltages from 1.3 V to 0.6 V, showing a change in the sign of the ionic net charge at both interfaces near 0.9 V. At any position with fewer cations than 10^17^ cm^−3^ – which corresponds to the immobile anion density in the simulation – a negative net charge density results.

We examine the deviation of the estimated bulk electric field (bias difference/*d*_bulk_) from the actual simulated value in the centre of the bulk, labelled as a bulk electric field correction factor (as calculated in Table 1 for a single BACE measurement). For the full set of BACE measurements (Fig. 5c), there is a significant difference when preconditioning is performed at 1.1 V. In Fig. 5d (black squares), we have summarised correction factors from simulations with preconditioning from 1.3 V to 0.6 V, followed by a switch to 0.25 V. There is a clear transition around the structural built-in potential of 0.9 V. The simulation results suggest that the potential drop across TLs for the base model increases significantly for preconditioning below 0.9 V. This occurs because the screened bulk electric field induces an additional potential drop across the TLs as shown in Fig. 5e and f, where the potential drop across both TLs is significantly higher for the preconditioning at 0.6 V compared to 1.1 V (blue *vs.* red lines). The addition of significant doping to the TLs (∼10^20^ cm^−3^) prevents their depletion in the simulation and different preconditioning does not have a significant effect anymore on the potential drop. As a result, the correction factors in Fig. 5d (red dots) stay below 1.1. The relevance of TL depletion agrees with a recent study investigating potential losses in perovskite device TLs, where the TL carrier depletion in operating devices was found to be caused by the formation of a space charge region within the thin TL due to the Schottky-type heterojunction formed with the transparent conductive oxide.^23^ The simulations of the base and high TL doping model in Fig. 5e and f are in good agreement with the simulation results in the study. Simulations without ions (Fig. 5e and f, black and green) are not fundamentally different, suggesting that ions are not the origin of TL depletion. Also, the effect on the stabilised efficiency may be small as the maximum power point is close to the built-in potential. However, ion-induced field screening alters the potential losses and carrier depletion in TLs depending on the preconditioning, which increases hysteresis.

Although the model matches experimental data, this hypothesis requires experimental verification, such as by changing transport layer doping. At this point, the results indicate that preconditioning below and above the structural built-in potential results in a distinctly different situation. Since measuring ionic currents must occur below diode injection voltages, staying below the structural built-in potential for preconditioning ensures a more consistent device state and results in a more controlled parameter extraction.

## Conclusions

We have analysed the application of the BACE technique in perovskite solar cells for the case that the ion density is high enough to fully screen an applied electric field. Drift–diffusion simulations reveal that when the voltage is switched from preconditioning to extraction, leading to a bulk electric field, all ions in the material start to migrate. This continues until the ions accumulate at or deplete from the interface region and the bulk electric field is screened. The experimental initial current density and decay dynamics largely depend on the ion conductivity, which is the product of the ion density and the mobility. With the ion density independently measured from impedance spectroscopy, the ion mobility is obtained from the BACE conductivity. We explain important differences between BACE results for the low- and high-ion density case. For a high ion density, the current decay dynamics are independent of the difference between the preconditioning and extraction voltage, whereas the timescale of current decline in the low-ion density regime strongly depends on the preconditioning voltage. The insight of this work qualifies BACE as an experimental method for the study of mixed ionic electronic perovskite materials beyond ion density extraction and sets limits and conditions upon its use to fully quantify ionic mobility and density in perovskite solar cell absorber layers.

## Data availability

The data supporting this article have been included as part of the ESI.†

## Conflicts of interest

H. J. S. is co-founder and CSO of Oxford PV Ltd.

## Supplementary Material

EE-018-D4EE02494J-s001
