# Supplementary material for: Probing ionic conductivity and electric field screening in perovskite solar cells: a novel exploration through ion drift currents
Source: Energy Environ Sci. 2024 Dec 17;18(3):1385–97. doi: 10.1039/d4ee02494j (PMC11667747; doi:10.1039/d4ee02494j)
Supplement: EE-018-D4EE02494J-s001 [file EE-018-D4EE02494J-s001.pdf]

Supplementary Information (SI) for Energy & Environmental Science.
This journal is © The Royal Society of Chemistry 2024

Supplementary Information

Probing ionic conductivity and electric field screening in perovskite solar cells: A novel exploration through ion drift currents

Matthias Diethelm,*[a] Tino Lukas,[a] Joel Smith,[a] Akash Dasgupta,[a] Pietro Caprioglio,[a] Moritz Futscher,[b] Roland Hany[c] and Henry J. Snaith*[a]

* mat.diethelm@gmail.com and henry.snaith@physics.ox.ac.uk

a. Department of Physics, University of Oxford, Clarendon Laboratory, Oxford OX1 3PU, UK

b. Empa, Swiss Federal Laboratories for Materials Science and Technology, Laboratory for Thin Films and Photovoltaics, CH-8600 Dübendorf, Switzerland

c. Empa, Swiss Federal Laboratories for Materials Science and Technology, Laboratory for Functional Polymers, CH-8600 Dübendorf, Switzerland.

Device fabrication

Below, the fabrication similar to reference [1] is described for the I:Br=75:25 device with the structure fluorine-doped tin oxide (FTO)/ tetrafluoro-7,7,8,8-tetracyanoquinodimethane (F4-TCNQ) doped poly(4-butylphenyl-diphenylamine) (PolyTPD)/ aluminium oxide nanoparticle (Al_2O_3)/ perovskite absorber with the ratio $Cs_{0.17}FA_{0.83}Pb(I_{0.77}Br_{0.23})_3$ (cesium (Cs), formamidinium (FA), lead (Pb), iodide (I), bromide (Br)) / phenyl-C_{61}-butyric acid methyl ester (PCBM)/ bathocuproine (BCP) /chromium (Cr)/ gold (Au).

The I:Br=90:10 device contains additionally the ionic additive 1-butyl-1-methylpiperidinium tetrafluoroborate $[BMP]^+[BF4]^-$ in the structure FTO/PolyTPD:F4-TCNQ/Al_2O_3/$Cs_{0.17}FA_{0.83}Pb(I_{0.9}Br_{0.1})_3$:$[BMP]^+[BF4]^-$/PCBM/BCP/Cr/Au.

The I:Br=75:25 SAM device contains the self-assembled monolayer (SAM) ([4-(3,6-dimethyl-9H-carbazol-9-yl)butyl]phosphonic acid) (Me-4PACz) within the structure FTO/Me-4PACz/Al_2O_3/$Cs_{0.17}FA_{0.83}Pb(I_{0.750}Br_{0.25})_3$/PCBM/BCP/Au as used in reference [2].

The I:Br=75:25 device was measured 3 weeks after fabrication. The I:Br=90:10 device was measured three months after fabrication, and the maximum power point tracking (MPPT)

efficiency remained constant during storage (18.06% after fabrication, and 18.0% prior to the manuscript measurement set). Furthermore, the ionic timescale also remained unchanged (comparable measurements to Fig. 1b, main text, after fabrication showed the same start of current decrease at 1ms and levelling off at 1s). The I:Br=75:25 SAM device was stored in a nitrogen filled glovebox for 13 months prior to the measurement. The cell exhibits a power conversion efficiency (PCE) of 16.37 % and a MPPT efficiency of 16.0 % after fabrication, and a PCE of 16.1 % and MPPT efficiency of 15.5% prior to the measurement for this work.

All materials were used as received without further purification. The perovskite precursor solutions were mixed from cesium iodide (CsI, 99.99%, Alfa-Aesar), formamidinium iodide (FAI, 99.99%, Dyenamo), lead iodide (PbI_2, 99.99%, TCI) and lead bromide ($PbBr_2$, ≥98%, Thermo scientific) with the stochiometry $Cs_{0.17}FA_{0.83}Pb(I_{0.77}Br_{0.23})_3$. The precursors were dissolved in anhydrous N,N-dimethylformamide (DMF, Sigma-Aldrich) and anhydrous dimethyl sulfoxide (DMSO, Sigma-Aldrich) at the volume ratio of DMF:DMSO = 4:1 and a concentration of 1.45 M. PolyTPD (1-Material) was dissolved in toluene (Sigma-Aldrich) in a concentration of 1 mg/ml along with 20 wt% of F4-TCNQ (Lumtec). PCBM (Solenne BV, 99.5%,) was dissolved in chlorobenzene (CB, Sigma-Aldrich) and 1,2-dichlorobenzene (DCB, Sigma-Aldrich) (CB : DCB = 3:1 in volume) and BCP (Xi'an Polymer, 99%) in isopropanol (IPA, Sigma-Aldrich) at a concentration of 20 and 0.5 mg/ml, respectively. The solutions were stirred overnight in a nitrogen-filled glovebox, PolyTPD:F4-TCNQ and BCP were stirred at 80 °C. Nanoparticle Al_2O_3 (<50 nm particle size, Sigma-Aldrich) solution was dissolved in isopropanol (IPA, Sigma-Aldrich) at a volume ratio of 1:150 just before spin coating. All solutions were filtered before use with a PTFE 0.2 µm filter.

FTO coated glass (Pilkington TEC 7, 7Ω/□) was patterned by etching with zinc powder and 2 M HCl. The substrates were cleaned in ultrasonic cleaning baths at 60 °C for 15 min in deionized water with 3.3% v/v solution of Decon 90 cleaning detergent; deionized water; acetone and IPA. After drying with dry nitrogen the substrates were treated with UV-Ozone for 15 min. 100 µL PolyTPD:F4-TCNQ was deposited dynamically at 2000 rpm for 20 sec and annealed at 130 °C for 5 min in ambient air. 400 µL Al_2O_3 nanoparticle solution was deposited inside a nitrogen-filled glove box at 6000 rpm for 30 s (not dynamically, 2000 rpm/s) and dried

at 100 °C for 2 min. The perovskite layer was spin coated dynamically with the following process: In a first step at 1000 rpm for 15 sec (200 rpm/s) 200 µL of the perovskite solution was dropped after 10 s, then at 5000 rpm (800 rpm/s) for 40 sec 200 µL anisole was dropped 10 s before the end to solvent-quench. The samples were annealed at 100 °C for 30 min to form the perovskite layer. 50 µL PCBM solution was dynamically dropped onto the perovskite layer at 2000 rpm for 20 s and annealed at 100 °C for 5 min. 100 µL BCP solution was dynamically coated onto the PCBM layer at 4000 rpm for 20 s and annealed at 100 °C for 2 min. Cr (3.5 nm) and Au electrodes (100 nm) were evaporated in a thermal evaporator (Nano 36, Kurt J. Lesker) placed in ambient environment.

Characterisation

All characterisation methods were performed on perovskite solar cells with an area of 0.25 cm^2. Bias-assisted charge extraction (BACE), impedance spectroscopy and current density-voltage (J-V) curves were performed sequentially with the Autolab (PGSTAT302N) directly after a calibrated power conversion efficiency and a steady-state power measurement in a Wavelabs SINUS-220 solar simulator.

BACE measurements were performed on the Autolab (ADC10M module) with a 60 s preconditioning bias at either 1.1 V, 0.6 V, 0 V or -0.2 V, followed by a switch to either -0.25 V, 0 V, 0.25 V or 0.5 V. Between each BACE measurement, the device was kept at open-circuit in the dark for 5 min to ensure full relaxation of the ions. The extracted ion density was calculated by integrating the current transient to the point where the current level dropped to 1%. The preconditioning duration of 60 s was chosen to achieve device stabilisation (see Fig. S2c). Switching times between different biases were less than 100 µs.

Impedance spectroscopy was performed on the Autolab (FRA32M module) at 0 V or 0.3 V with an alternating 20 mV amplitude signal at several frequencies.

Current density-voltage (J-V) hysteresis measurement at several speeds were performed on the Autolab by calibrating the intensity of the warm white LED with (AUT.LED.LDCWW.S, ca.

410 nm- 770 nm) to get the same short-circuit current as resulted from the Wavelabs SINUS-220 solar simulator measurement described below.

Power conversion efficiency from current density-voltage (J–V) curves and maximum power point tracking (MPPT) efficiency were taken using a Keithley 2400 source measure unit (SMU) in ambient air. These measurements were conducted under light (simulated AM1.5 G irradiance produced by a Wavelabs SINUS-220 solar simulator), where the intensity was calibrated with a certified KG3-filtered reference diode (Fraunhofer). The solar cell's active area was covered with a black-anodised metal aperture to 0.25 cm^2. The J–V scans were taken from 1.2 V (forward bias) to -0.2 V, and from -0.2 V to 1.1 V, both at a scan rate of 0.33 Vs^{-1}. MPPT was carried out using a gradient descent algorithm to determine the steady-state power conversion efficiency.

External quantum efficiency (EQE) spectra were obtained using a chopped halogen lamp light source in conjunction with a monochromator, calibrated using a certified Si reference diode.

Time-Correlated Single Photon Counting was performed on the Fluo Time 300 using a pulsed laser at 634 nm, with a repetition rate of 1 MHz. The timing was electronically managed using a TimeHarp 260. The PL decays were recorded at the peak wavelength of the PL spectra.

Drift-diffusion simulations were done with Driftfusion[3,4], available at https://github.com/barnesgroupICL/Driftfusion. The Supplementary Information provides further simulation details.

Supplementary Information Figures and Notes

In the order of their appearance in the main text.

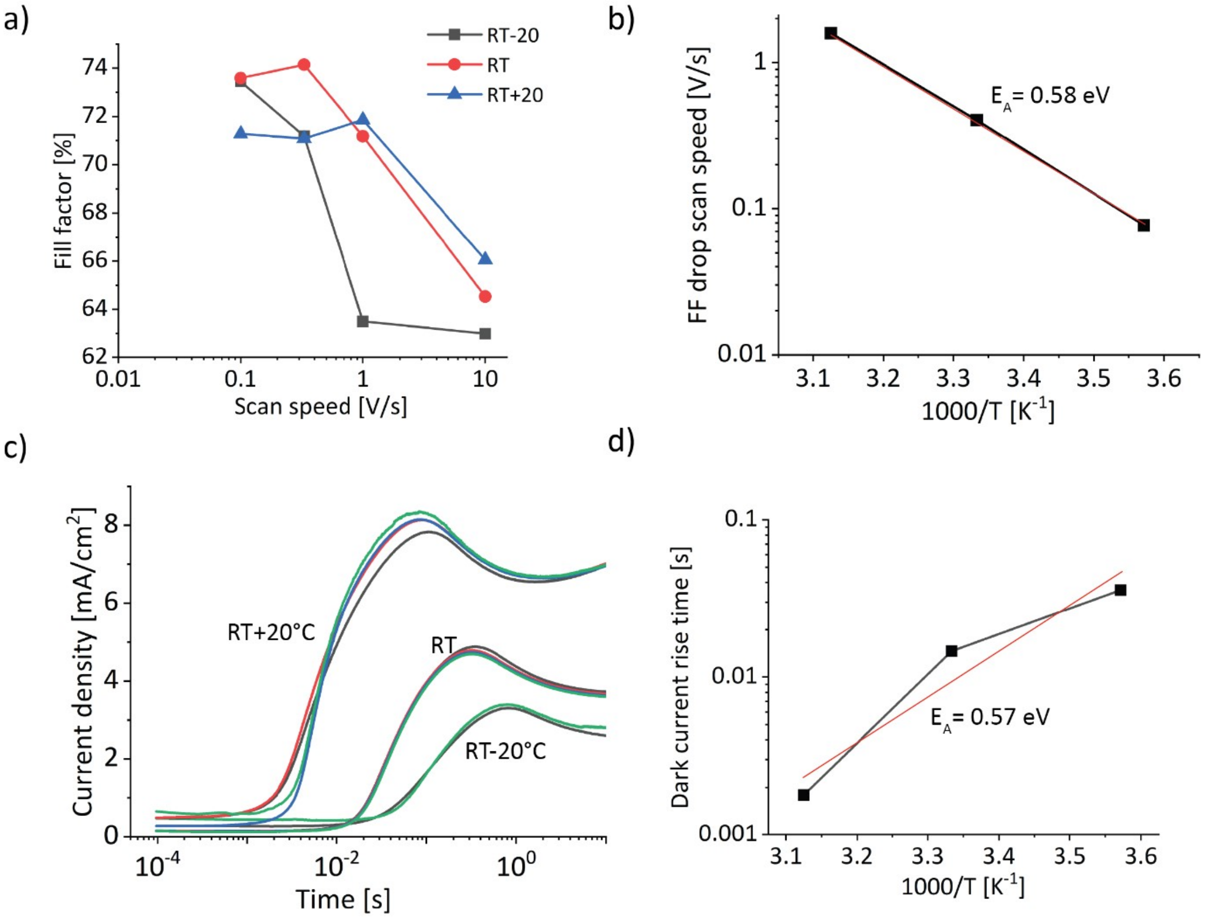

Fig. S1 Temperature-dependent measurements at room temperature (RT), RT+20 °C and RT-20 °C on the I:Br=75:25 device. The activation energy E_A is extracted in b) from the temperature-dependent change in scan speed where the fill factor starts to decrease in a), and in d) from temperature-dependent changes of the time when the dark current increases in c) when the bias is switched to 1.15 V after a preconditioning at 0 V. Both show a $\propto \exp\left(\frac{E_A}{k_B T}\right)$ dependency (k_B: Boltzmann constant, T: Temperature). Hysteresis, as shown here by changes in fill factor, has been attributed to ionic dynamics. An increase in dark current is related to a decrease in the interface barrier when ions move away from the interface.[5]

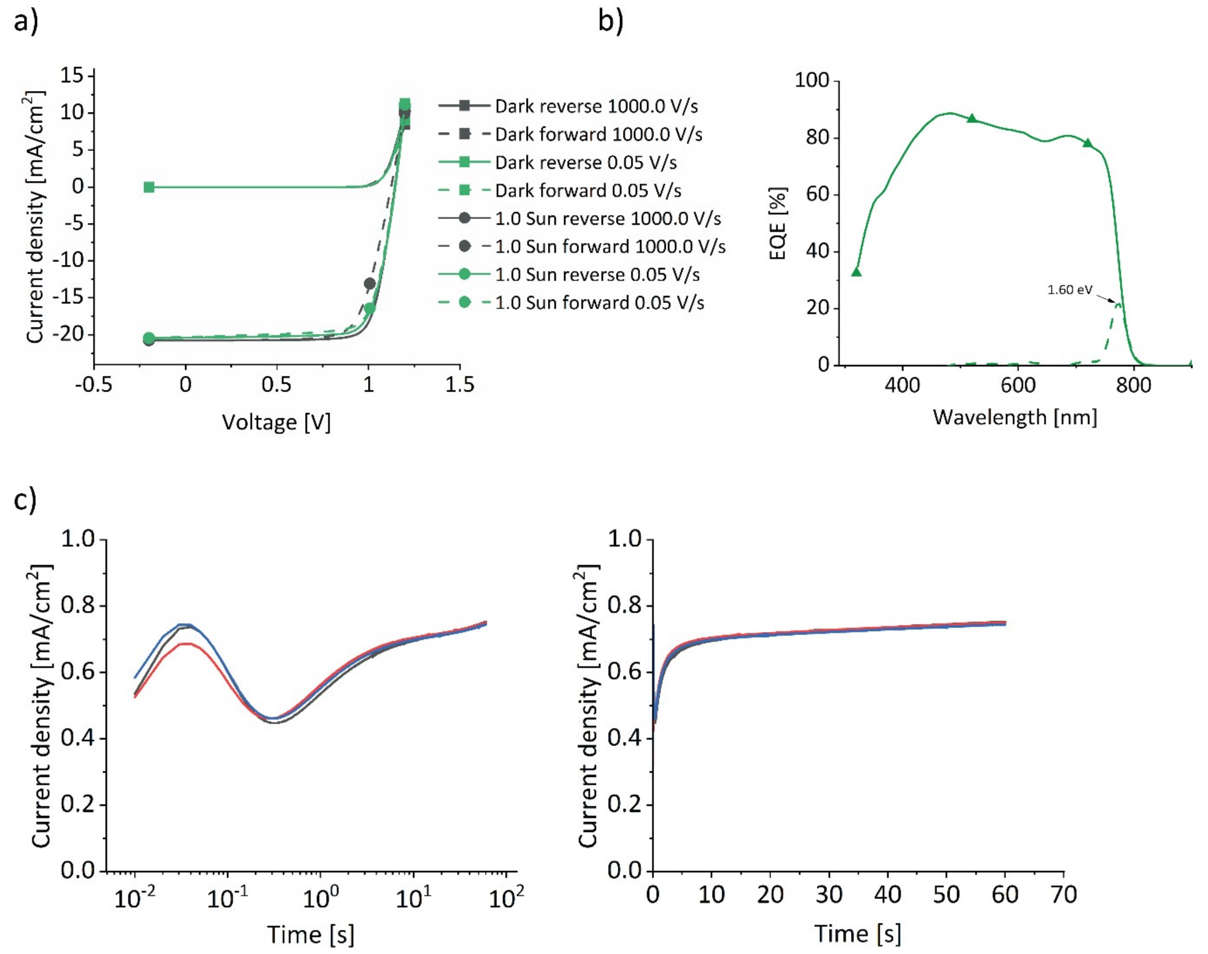

Fig. S2 I:Br=90:10 device measurements. a) Current-voltage hysteresis measurements at different scan speeds. b) External quantum efficiency measurement with indicated bandgap, calculated from the peak of the derivative dEQE/dλ. c) 60 s preconditioning measurement at 1.1V of the measurement in Fig. 1b (green). The largest changes occur on timescales up to seconds, as shown in the logarithmic scale on the left. After 10 s the current remains almost constant, as better seen in the linear scale on the right.

Supplementary Note SN1

To ensure full relaxation of the ions after the BACE measurement, the rest time at open-circuit between measurements was chosen 10 times longer than the transient measurement length. Slow ion relaxation is reported in literature and has been explained by trapping of ions at grain boundaries into a potential energy minima[6] and adsorption/attachment to the interface.[7,8] Phase segregation in mixed halide systems tends to get slower with higher iodide content, where PL changes are observed in timescales of minutes for $I_{0.5}Br_{0.5}$, and hours for $I_{0.6}Br_{0.4}$.[9–12]

It is therefore expected that the investigated $I_{0.77}Br_{0.23}$ and $I_{0.9}Br_{0.1}$ systems do not show halide segregation observable in the PL in the measurement timescale of seconds. This is shown in Fig. Note SN1a, where no decline of peak intensity (Fig. Note SN1b) measured at 750 nm at an excitation laser wavelength of 634 nm with a fluence of $5*10^{18}$ photons s^{-1} cm^{-2} or 16000 W/m^2 within 10 s is observable. The peak PL is expected to drop drastically during phase segregation.[11]

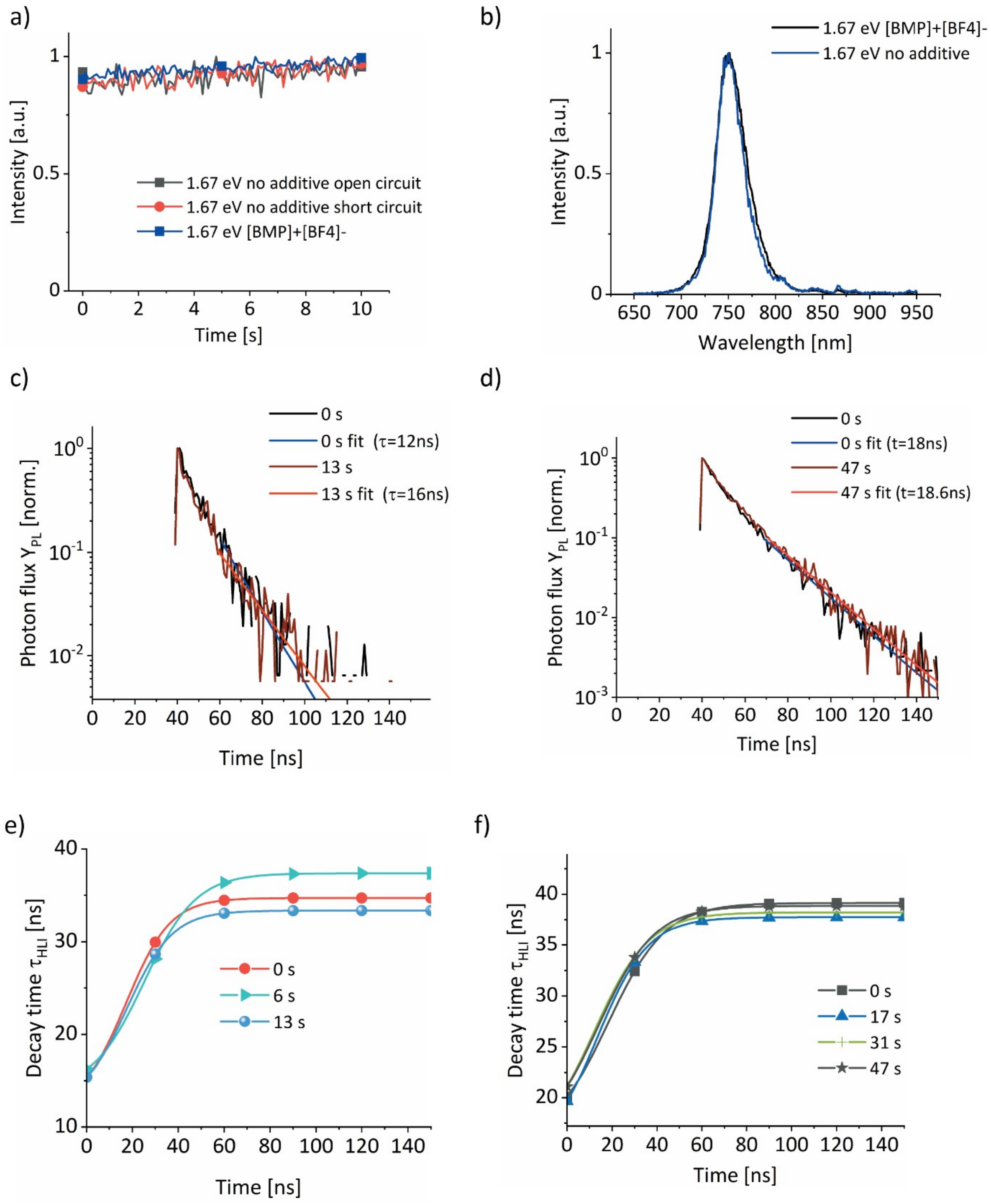

Fig. Note SN1 I:Br=75:25 device measurements. a) No decline of peak intensity measured at 750 nm at an excitation laser wavelength of 634 nm with a flux of $5*10^{18}$ photons s^{-1} cm^{-2} or 16000 W/m^2 within 10 s. b) PL measurements. c) and e) are measurements on a device without additive, d) and f) are measurements on a device with additive. c-f) same conditions as in a). Analysis e) and f) is calculated by differentiating the TRPL measurement to get the high-level

injection differential lifetime $\tau_{HLI}(t) = -\frac{1}{\frac{dln(Y_{PL}(t))}{dt}}$.[13] Note the time resolution is not fast enough to observe changes within the first second and therefore the ion induced changes in recombination.

Supplementary Note SN2

The radiative dark recombination current density $J_{0,rad}$ for a material with a bandgap of 1.66 eV is in the order of 10^{-19} $\mu A/cm^2$, calculated from the integral of the blackbody flux times the external photovoltaic quantum efficiency. J_0 is then calculated from the external electroluminescence quantum efficiency $EQE_{EL} = \frac{J_{0,rad}}{J_0} = \frac{J_{0,rad}}{J_{0,rad} + J_{0,non-rad}}$. With an EQE_{EL} of 10^{-8}, J_0 is estimated to be 10^{-11} $\mu A/cm^2$, which is much smaller than the measured current densities of $\approx 10^0$ $\mu A/cm^2$.[14]

Typically, the trap dynamics for electronic charges is very fast. According to the few reports on trap capture dynamics in perovskite devices, the capture coefficients for free electrons and free holes can be as high as 10^{-10} cm^3 s^{-1} in illuminated samples, leading to sub-µs timescale for recombination currents.[15,16] Trapped charges can lead to slow transients if the only pathway is emission across an energy barrier, which can occur when an operated solar cell with filled trap states is switch to dark at a bias below injection. The timescale depends then exponentially on the energy barrier and can be in the order of ms to s.[17] However, the device shows a correlation between the device response timescales under both light and dark conditions. This implies that a process other than trap recombination dynamics might be involved. Trap recombination can only explain a slower timescale above milliseconds in dark conditions, but it doesn't account for the timescale in illuminated conditions. Therefore, it's probable that a different process is at play here, one that can also explain the slow timescale when the device is exposed to light.

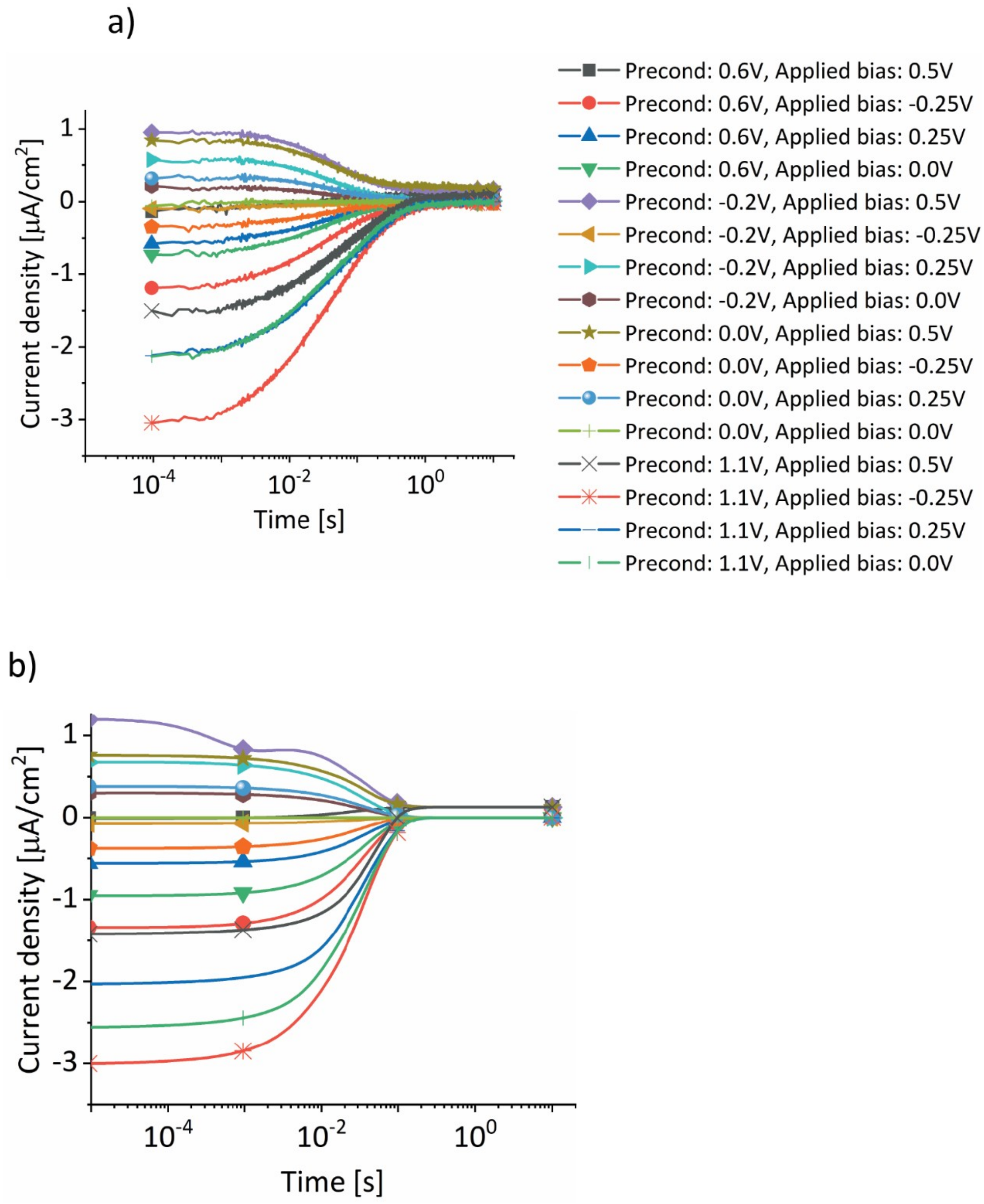

Fig. S3 a) Full current trends for data shown in Fig. 1d and 1e, main text. b) Simulated current trends using the experimental measurement protocol.

Supplementary Note SN3

Starting point simulation parameters were internal published SCAPS and SETFOS model parameters.[18,19] Energy levels, electronic mobilities, recombination in the bulk and at the interfaces were slightly adjusted to match the recombination-sensitive measurements in Fig. S10

SN3 qualitatively, specifically those of the short-circuit current (J_{SC}) and open-circuit voltage (V_{OC}). Ion density and mobility were altered to quantitatively fit the BACE measurements.

	PolyTPD	Perovskite	PCBM
Layer thickness [cm]	$1*10^{-6}$	$5*10^{-5}$	$3*10^{-6}$
Electron affinity [eV]	-2.3	-3.8	-3.9
Ionisation potential [eV]	-5.1	-5.36	-6.05
Equilibrium Fermi level [eV]	-4<br>Fig 5d: -5.05	-4.63	-5<br>Fig 5d: -3.95
SRH trap energy [eV]	-4.3	-4.6	-5
Conduction band effective density of states [cm^{-3}]	$1*10^{20}$	$2.2*10^{18}$	$1*10^{20}$
Valence band effective density of states [cm^{-3}]	$1*10^{20}$	$2.2*10^{18}$	$1*10^{20}$
Intrinsic cation density [cm^{-3}]	-	$9.34*10^{16}$	-
Limiting cation density [cm^{-3}]	-	$1.21*10^{22}$	-
Electron mobility [cm^2 V^{-1} s^{-1}]	$1*10^{-4}$	10	$1*10^{-2}$
Hole mobility [cm^2 V^{-1} s^{-1}]	$1*10^{-4}$	10	$1*10^{-3}$
Cation mobility [cm^2 V^{-1} s^{-1}]	-	$1*10^{-8}$	-
Relative permittivity	4	23	5
Uniform generation rate [$cm^{-3\ s-1}$]	0	$2.64*10^{21}$	0
Band-to-band recombination rate coefficient [cm^{-3} s^{-1}]	0	$3.6*10^{-12}$	0

SRH electron lifetime [s]	$1*10^{-9}$	$5*10^{-8}$	$1*10^{-9}$
SRH hole lifetime [s]	$1*10^{-9}$	$5*10^{-8}$	$1*10^{-9}$

PolyTPD/perovskite interface

Electron surface recombination velocity (S_n): 2 cm s^{-1}, hole surface recombination velocity (S_p): 2 cm s^{-1}

Perovskite/PCBM interface

S_n: 2 cm s^{-1}, S_p: 2 cm s^{-1}

Electrode p-side next to PolyTPD

Equilibrium Fermi energy: -5 eV, S_n: $1*10^3$ cm s^{-1}, S_p: $1*10^3$ cm s^{-1}

Electrode n-side next to PCBM

Equilibrium Fermi energy: -4.1 eV, S_n: $1*10^3$ cm s^{-1}, S_p: $1*10^3$ cm s^{-1}

Complex refractive indices for I:Br=75:25 devices were taken from reference [20] and were shifted by 48 nm for I:Br=90:10 devices. This approximation is based on the absorption coefficient change shown in reference [21].

A low doping density in the PolyTPD layer was necessary to quantitatively better match the overestimated dark current increase at forward bias, even though the layer is doped with F4-TCNQ. An experimental reason could be that the F4-TCNQ is washed out during spin-coating of the next layer. However, it could also be that the transport layer is depleted by the TCO-induced space charge region, as recently studied in detail.[22]

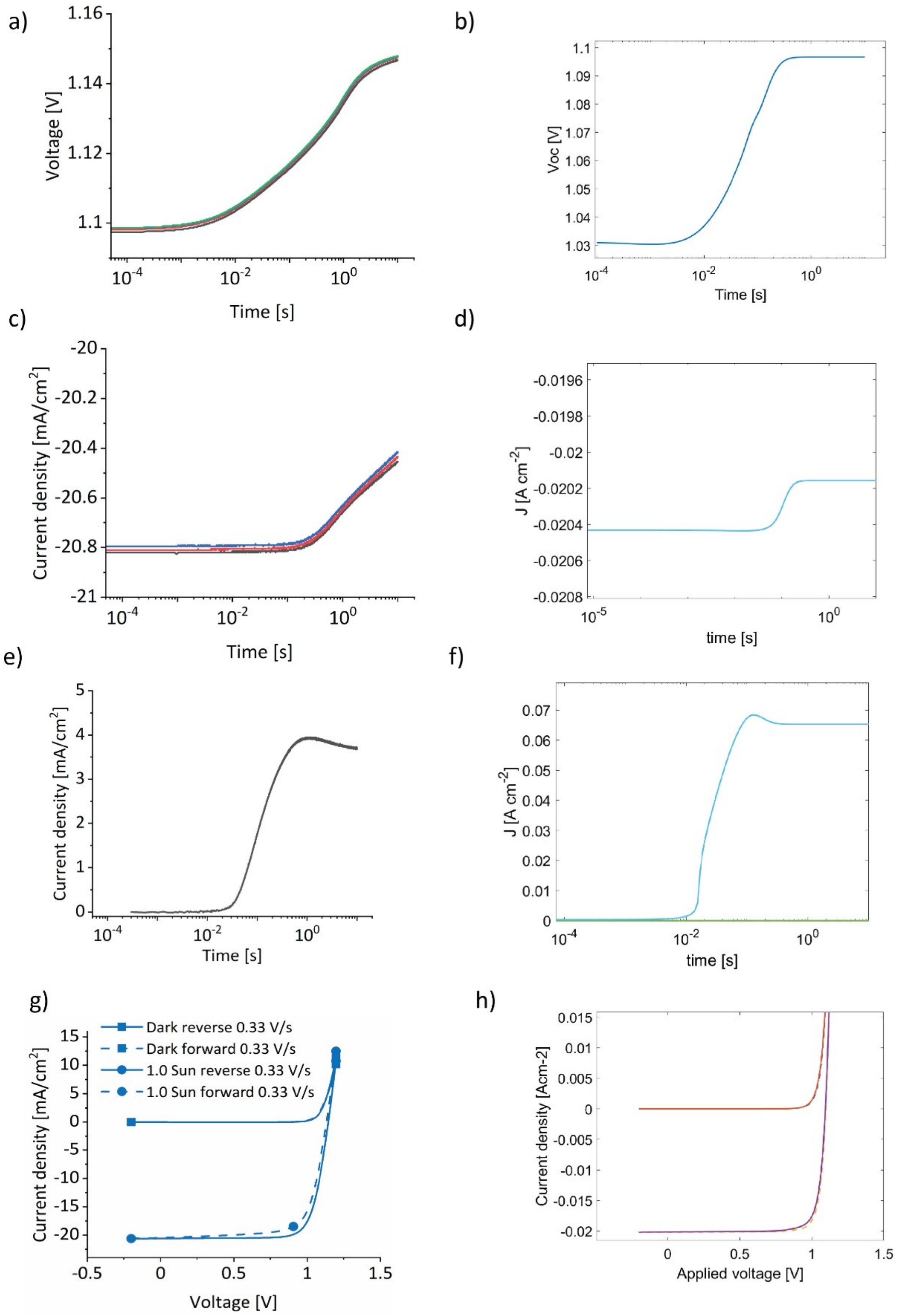

Fig. SN3 Characteristic measurements of the I:Br=90:10 device in light and in the dark (a,c,e,g) and corresponding simulations (b,d,f,h). a) V_{OC} increase after a preconditioning of 60 s at J_{SC}, c) J_{SC} decrease after a preconditioning of 60 s at V_{OC}, e) dark current after 60 s preconditioning at open-circuit in the dark, and g) J-V hysteresis measurements. Simulations were made as the measurements were performed.

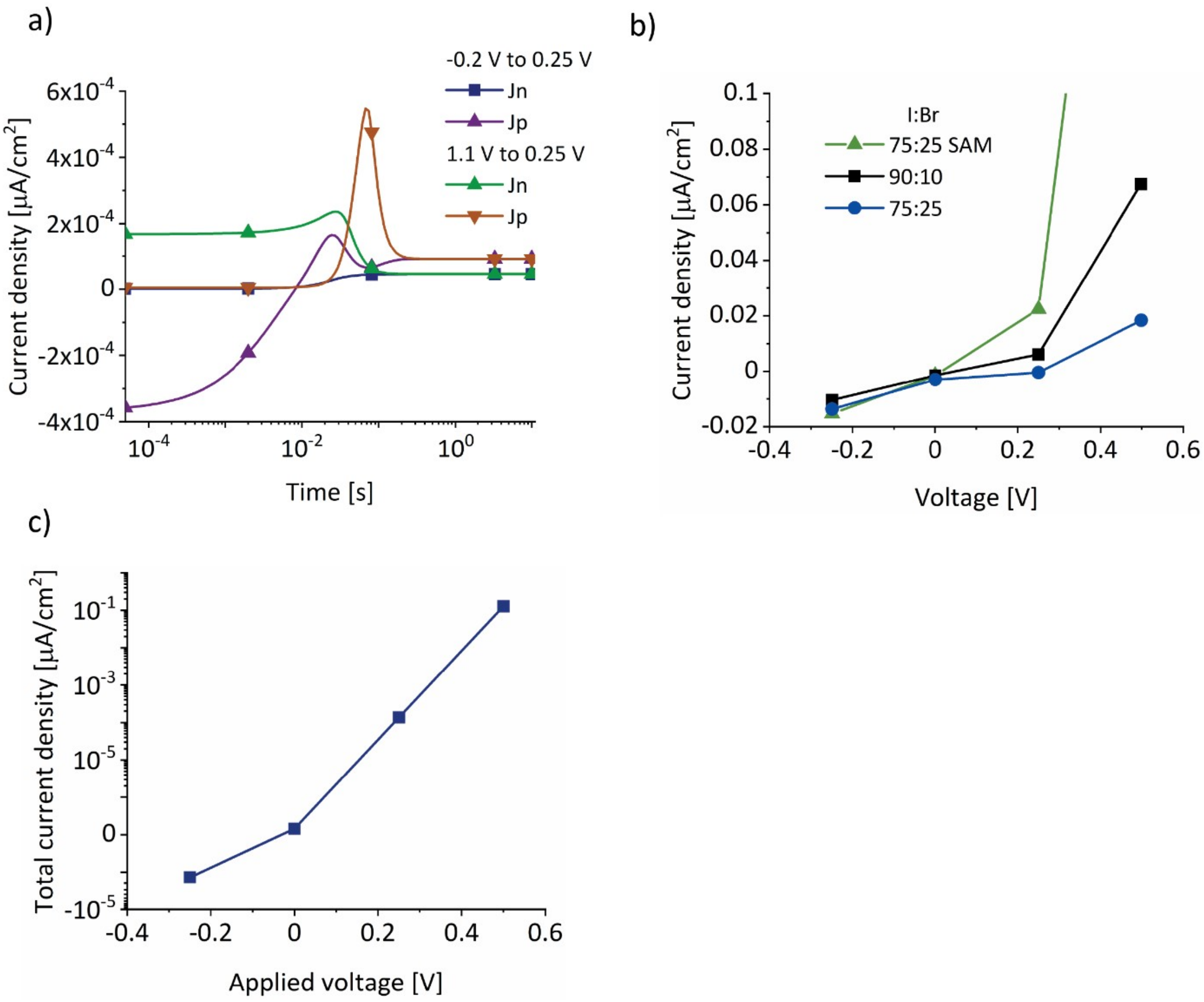

Fig. S4 a) Electron and hole currents of the simulation in Fig. 2a. b) Measured dark currents with integration time of 60 s at every applied voltage. The measured current at 0.5 V exhibits a rapid increase in current density from a linear region below 0.5 V. This suggests that forward current dominates the diode, which agrees with the simulated current density at 0.5 V in c). Simulated dark current. The experimental current below 0.5 V is much higher than in the simulation, which might be due to a shunt. Such low shunting currents do not have an influence on the operation of the solar cell, however.

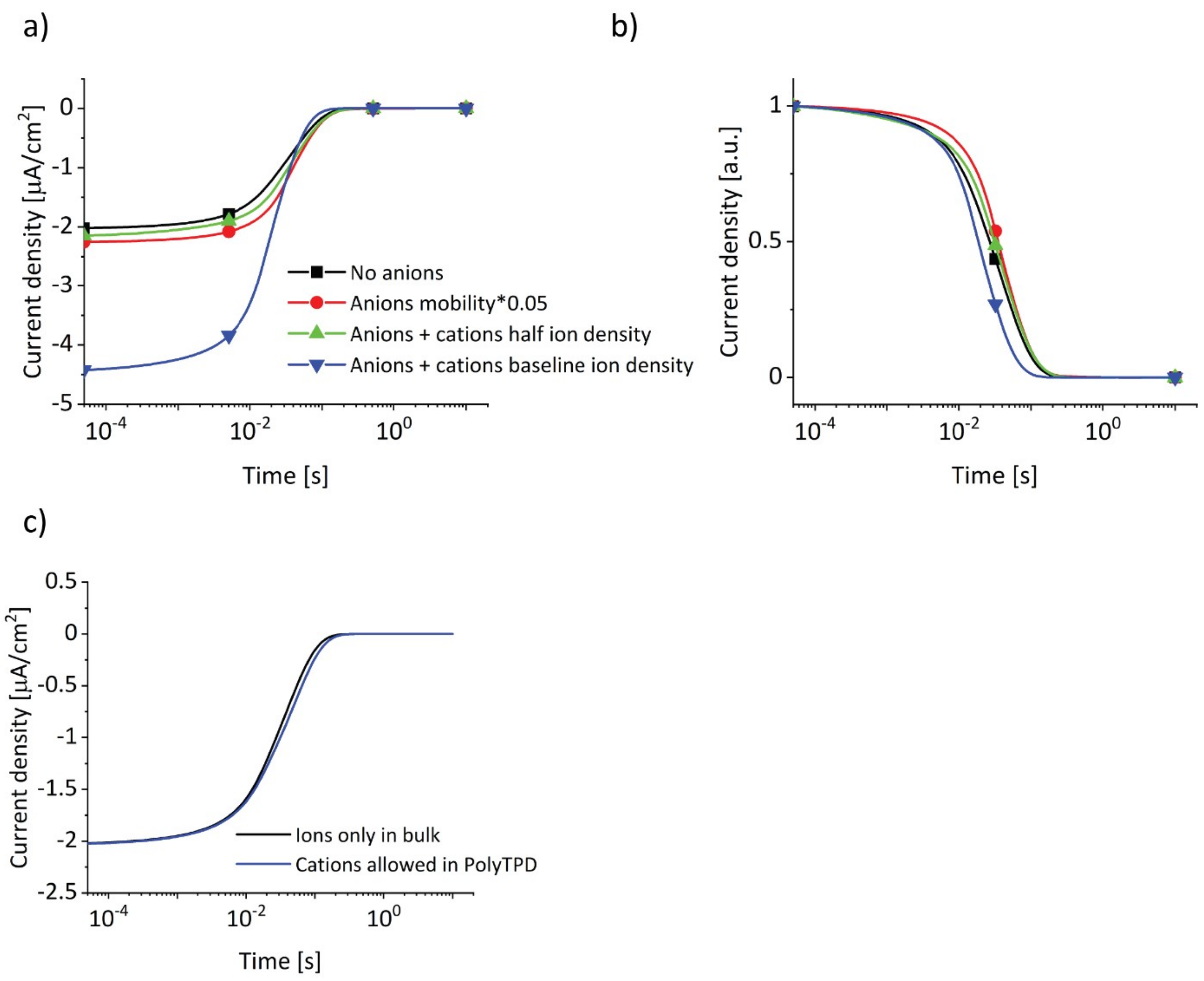

Fig. S5 a) BACE simulation curves (1.1 V → 0.25 V) when adding mobile anions. Black: $n_{cation} = 9 * 10^{16}\, cm^{-3} \equiv n_{c0},\ n_{anion} = 0,\ \mu_{cation} = 1 * 10^{-8}\, cm^2\, V^{-1}\, s^{-1} \equiv \mu_{c0}$; red: $n_{anion} = n_{cation} = n_{c0},\ \mu_{cation} = \mu_{anion} * 20 = \mu_{c0}$; green: $n_{anion} = n_{cation} = 0.5 * n_{c0},\ \mu_{cation} = \mu_{anion} = \mu_{c0}$; blue: $n_{anion} = n_{cation} = n_{c0},\ \mu_{cation} = \mu_{anion} = \mu_{c0}$. The influence of mobile anions becomes only apparent when the anionic conductivity, i.e. the product $n_{anion} * \mu_{anion}$ is comparable to the cationic conductivity. For example, the influence of a high anion density with a low mobility (red curve) is small. When anions and cations are present in similar concentrations and with similar mobilities (blue), the current levels doubles because of the overall ion conductivity increases by a factor of two. b) Normalized currents of simulations in a). c) BACE simulation (1.1 V → 0.25 V) with and without allowed movement of cations into the polyTPD layer, which is achieved by setting the anion mobility to $1 * 10^{-8}\, cm^2\, V^{-1}\, s^{-1}$ or zero in the transport layer.

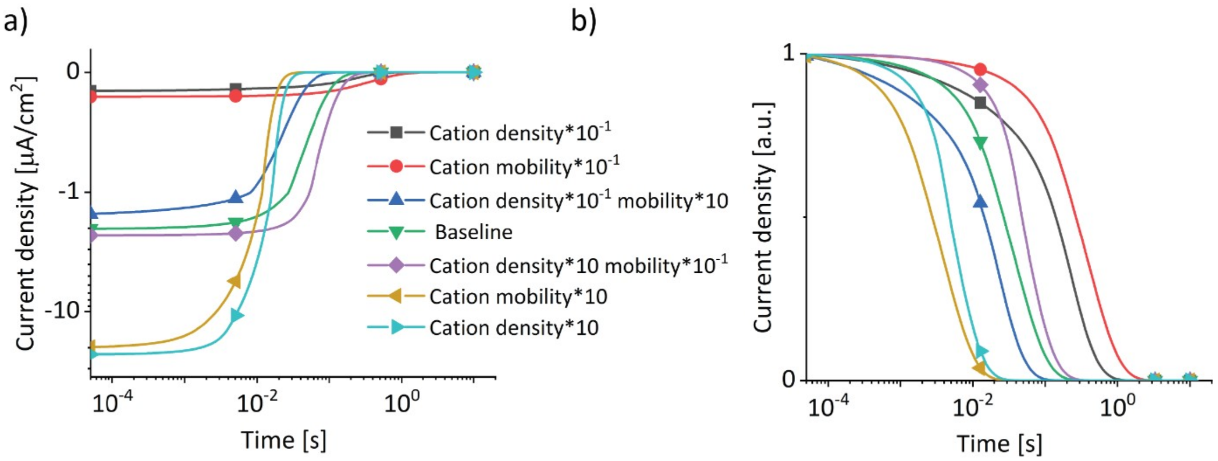

Fig. S6 a) BACE simulations (1.1 V → 0.25 V) with ion conductivity and/or mobility differences of one order of magnitude. For the baseline simulation, the cation density was $9*10^{16}$ cm^{-3}, the cation mobility $1*10^{-8}$ cm^2 V^{-1} s^{-1}. The ion conductivity (i.e. the mobility density product) dominates the current level, and also the timescale of the current decrease as shown in the normalized plots in b). The largest deviations in each of the three conductivity groups with conductivity $1.5*10^{-10}$ S/cm (blue, green, violet), $1.5*10^{-11}$ S/cm (black, red) and $1.5*10^{-9}$ S/cm (orange, light blue) are due to ion concentration-induced changes at the interfaces, which results in a different ionic interface capacitance[23] that changes the timescale of current decrease. Also displacement currents and potential losses in parts of the device other than the bulk differ slightly. This leads to slightly different correction factors, as explained in the Supplementary Note SN5.

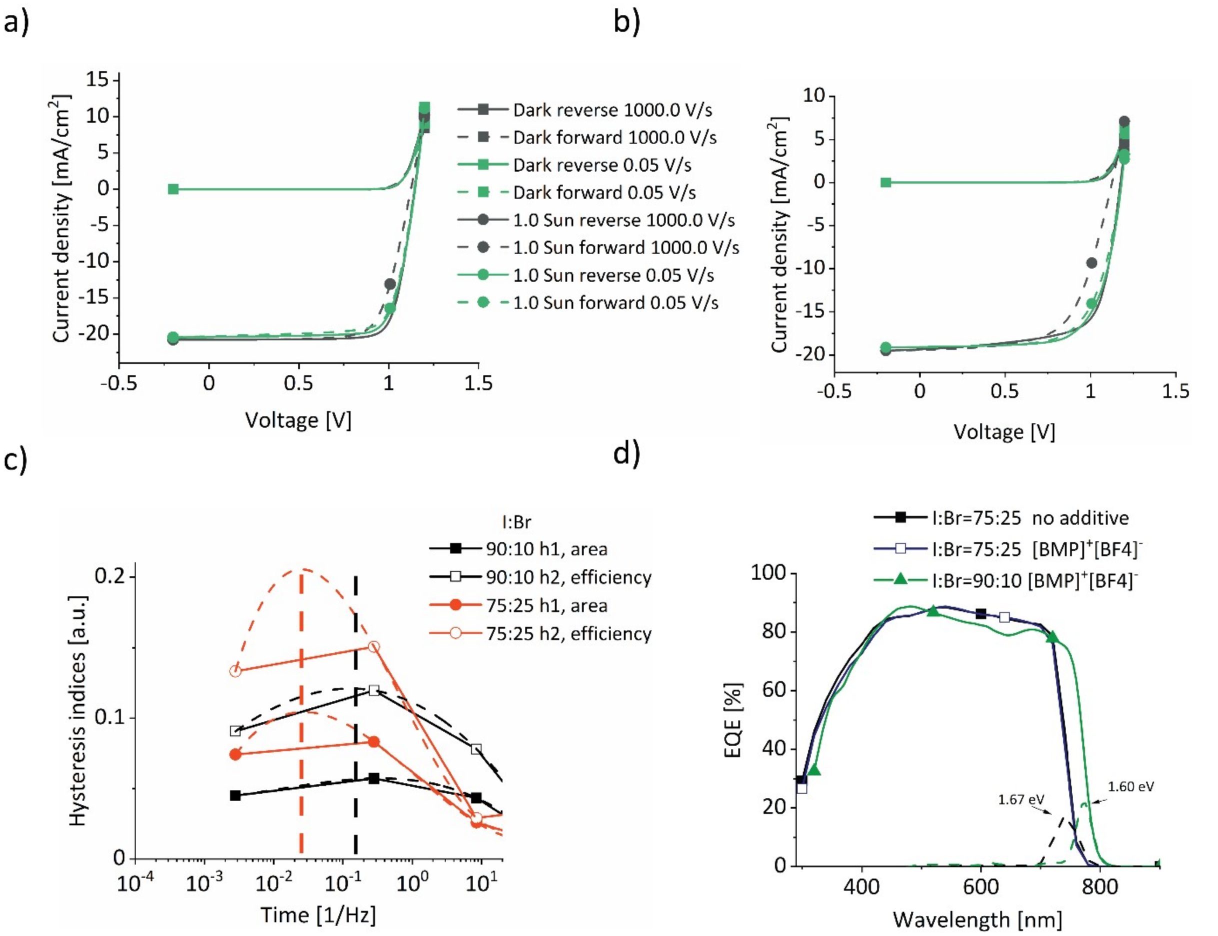

Fig. S7 J-V curves of the I:Br=90:10 device (a) and the I:Br=75:25 device (b). c) Different hysteresis index as defined in reference [24]. Indices h1 and h2 are computed by dividing the area and efficiency, respectively, of the forward J-V measurement by the reverse J-V measurement. The estimated peak shift (dashed lines) of the hysteresis aligns with the BACE measurement time shown in Fig. 3c, main text. d) The external quantum efficiency measurement is presented with the indicated bandgap, which is calculated from the peak of the derivative dEQE/dλ.

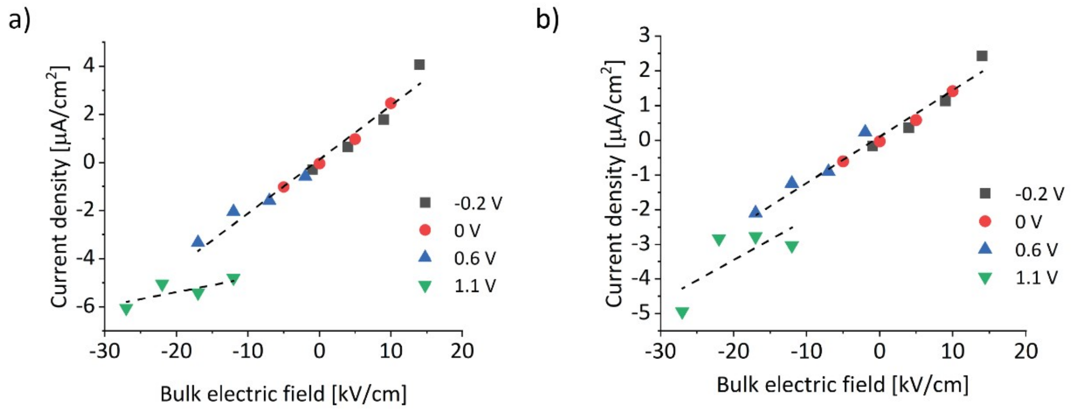

Fig. S8 Measurements of Fig. 1 (main text) were repeated with a I:Br=75:25 device (a) and a I:Br=75:25 device using a SAM layer (b). Dashed lines are linear fits of the data, the slope of which gives the conductivity (indicated as stars in Fig. 3b, main text).

Supplementary Note SN4

With a transient ion drift (TID) experiment the ion density can be extracted from a change in capacitance as a result of ionic layers at the interface when the bias voltage is switched at high frequencies.[25] It is not clear, however, whether the method is suited for the high ion density present in our perovskite films. TID uses a model which was developed for measuring impurities in silicon[26] and was later applied to second generation solar cell materials, [27] where the ion density is lower than the doping density. This is not the case in a perovskite material where the ion density is high, but the doping density is low.[28–30]

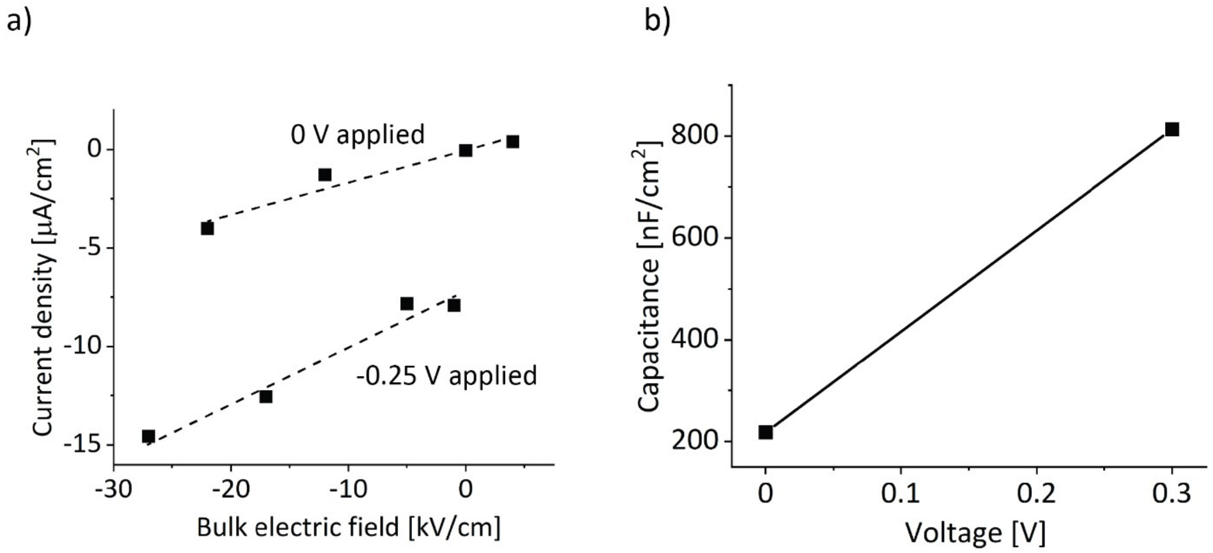

Fig. S9 a) BACE data for a 75:25 device, where conductivity is extracted from the slope (multiplied by 10^{-9} to convert the unit from nS/cm to S/cm), here $1.6*10^{-10}$ S/cm for the 0 V applied bias and $2.8*10^{-10}$ for the -0.25 V applied bias, on average $2.2*10^{-10}$ S/cm. The device exhibited significant shunting behaviour. As a result, the applied -0.25 V measurements, when compared to the 0 V measurements, showed an additional negative shunt current on top of the ion drift current for each preconditioning. However, since the shunt current is constant at a certain applied bias, the conductivity can still be extracted. b) Impedance spectroscopy at 0.1 Hz in the dark at 0 V and 0.3 V. The ion density is calculated from the Mott-Schottky equation applied to the case of interface capacitance $\frac{d\left(C_{ion}^{-2}\right)}{dV_{applied}}=-\frac{2}{n_{ion}q\varepsilon_0\varepsilon_r}$.[23]

Supplementary Note SN5

The BACE simulations and measurements in Fig. 1e show the same current density behaviour for different BACE bias switches, therefore the extracted ion conductivity from the measurement and simulation are very similar, as shown in Table S1. However, when the actual ion conductivity in the simulation is computed using the formula $\sigma_{ion} = q * n_{ion} * \mu_{ion}$, based on the simulation parameters, the resulting conductivity is approximately twice as large as the ion conductivity derived from the BACE current simulation. This is because of two reasons. First, the bulk electric field screening, i.e. the change in the bulk electric field, results in a displacement current $J_D = \frac{\varepsilon_0\varepsilon_r}{A} * \frac{d}{dt}E$ as shown in Fig. Note SN5. This causes the actual ion drift current and therefore the ion conductivity to be underestimated, as the displacement current of -0.3 μA/cm² shifts the total measured current down to 0.7 μA/cm² compared to the drift current of 1 μA/cm². Therefore, the measured current needs to be corrected by a factor $1\frac{\mu A}{cm^2}/0.7\frac{\mu A}{cm^2} = 1.43$. Second, a voltage drop across the transport layers results in an overestimation of the bulk electric field, leading to an underestimation of the ion conductivity by 40%. The electric field in Fig. Note SN5 is 6.5 kV/cm, while from the bias switch from -0.2 V preconditioning to 0.25 V an electric field of 9 kV/cm is assumed for a thickness of 500 nm in the bulk. The correction factor here is therefore $9\frac{kV}{cm}/6.5\frac{kV}{cm} = 1.38$. Correction factors for all BACE measurements are discussed in Fig. 5c and Supplementary Note SN6)

Table S1 Comparison of extracted conductivity from measured and simulated ion drift BACE to simulation input parameter conductivity of the 90:10 device (fits from Fig. 1e)

Ion drift BACE measurement preconditioning <1.1V	Ion drift BACE measurement preconditioning 1.1V	Ion drift BACE simulation preconditioning <1.1V	Ion drift BACE simulation preconditioning 1.1V	Simulation
6.9E-11 S/cm	9.2E-11 S/cm	7.9E-11 S/cm	1.1E-10 S/cm	1.5E-10 S/cm

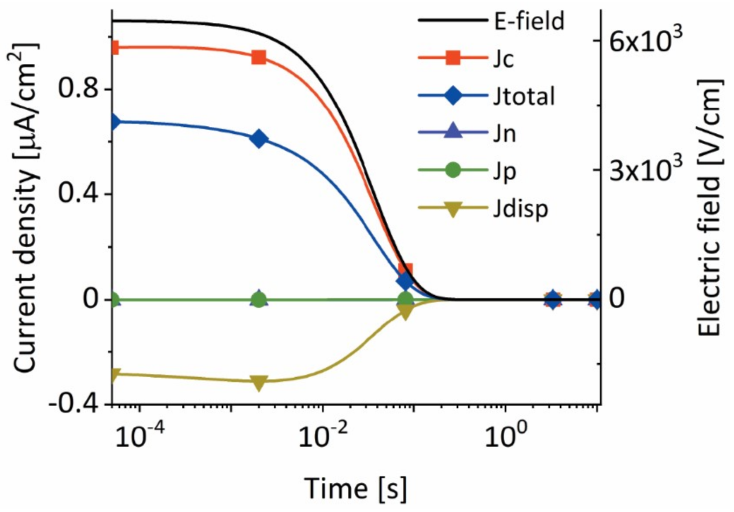

Fig. Note SN5 Drift-Diffusion simulation of Fig. 2a including displacement and electronic currents (-0.2 V preconditioning to 0.25 V applied bias).

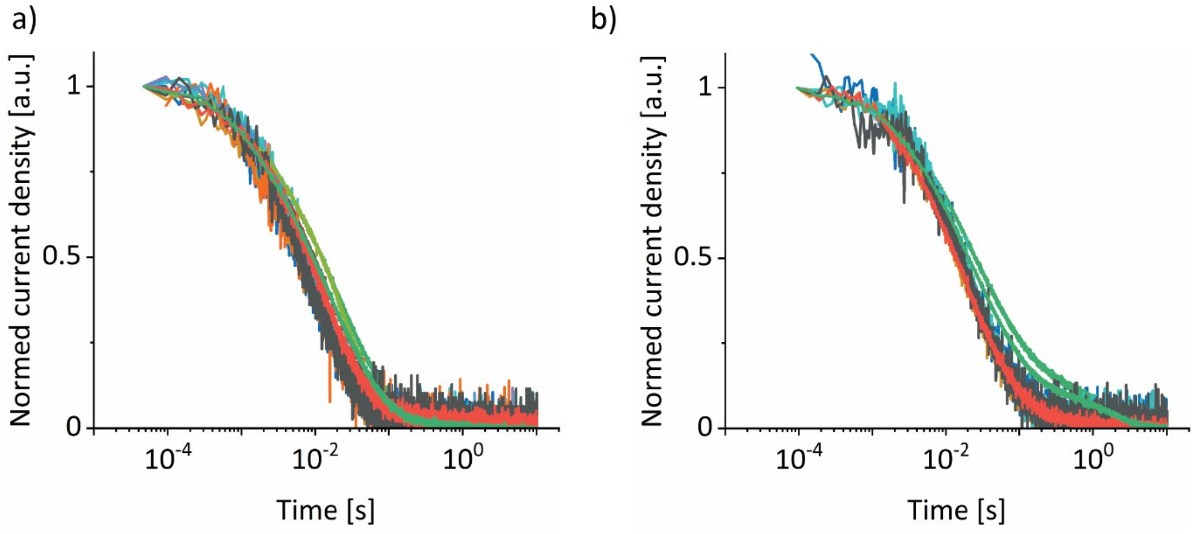

Fig. S10 Normalised BACE measurements used for Fig. S8 for a) I:Br=75:25 devices, b) I:Br=75:25 devices with a SAM layer.

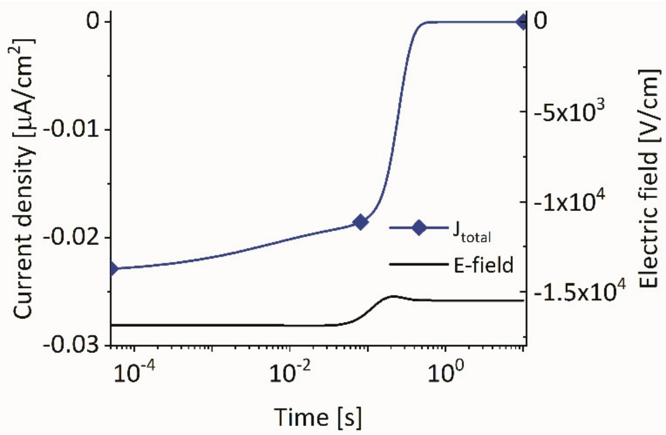

Fig. S11 The ion density in the model is reduced by two orders of magnitude to $1*10^{15}$ cm^{-3}, such that the bulk electric field is no longer fully screened. Only a small decrease in electric field is observed while the current density decreases to zero.

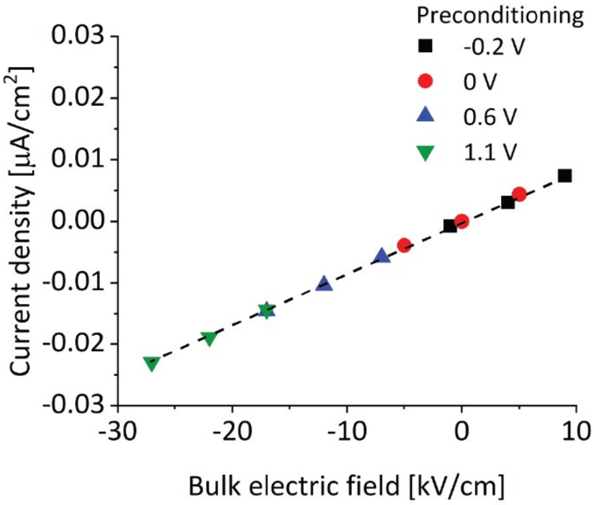

Fig. S12 Compilation of BACE simulations for the low ion density case. The current density levels at early times (<10^{-3} s) for 16 simulations for each combination of preconditioning (-0.2, 0, 0.6 and 1.1 V) and applied voltage (-0.25, 0, 0.25 and 0.5 V) are plotted against the bulk electric field. Before a voltage change, the ion drift and diffusion currents at the interface are balanced. Any voltage change, whether negative or positive, will result in a corresponding electric field change in the interface region, and thus ion drift current

(while at preconditioning ≤ 0.6 V the bulk is ion free and therefore shows no ion drift current in the bulk). The extracted conductivity is $8.48*10^{-13}$ S/cm.

Supplementary Note SN6

In the Supplementary Note SN5 necessary corrections for the measured current density and the estimated bulk electric field were discussed only for one BACE measurement. Here, we consider the dependence of these corrections on the preconditioning voltage, as for all device architectures a shift towards higher currents is observed for a preconditioning at 1.1 V. We note that this does not necessarily lead to a deviation in the conductivity as shown in Fig. Note SN6a, as the conductivity is extracted from the slope of multiple measurements at the same preconditioning. This appears to cancel out the shift towards higher currents.

On top of deviations in bulk electric field correction factor (Fig. 5c) for different preconditioning, the displacement current $J_D = \frac{\varepsilon_0 \varepsilon_r}{A} * \frac{d}{dt} E$ gives a very similar correction factor for all preconditioning conditions ≤ 0.6 V although the bulk electric field varies (Fig. SN6b). This is because both the ion drift current and the displacement current depend on the electric field, so both vary in the same way when the bulk field changes. This is also the reason why the two correction factors correlate very well, as shown for the simulations for preconditioning from 1.3 V to 0.6 V and a subsequent switch to 0.25 V (Fig. Note SN6c and d).

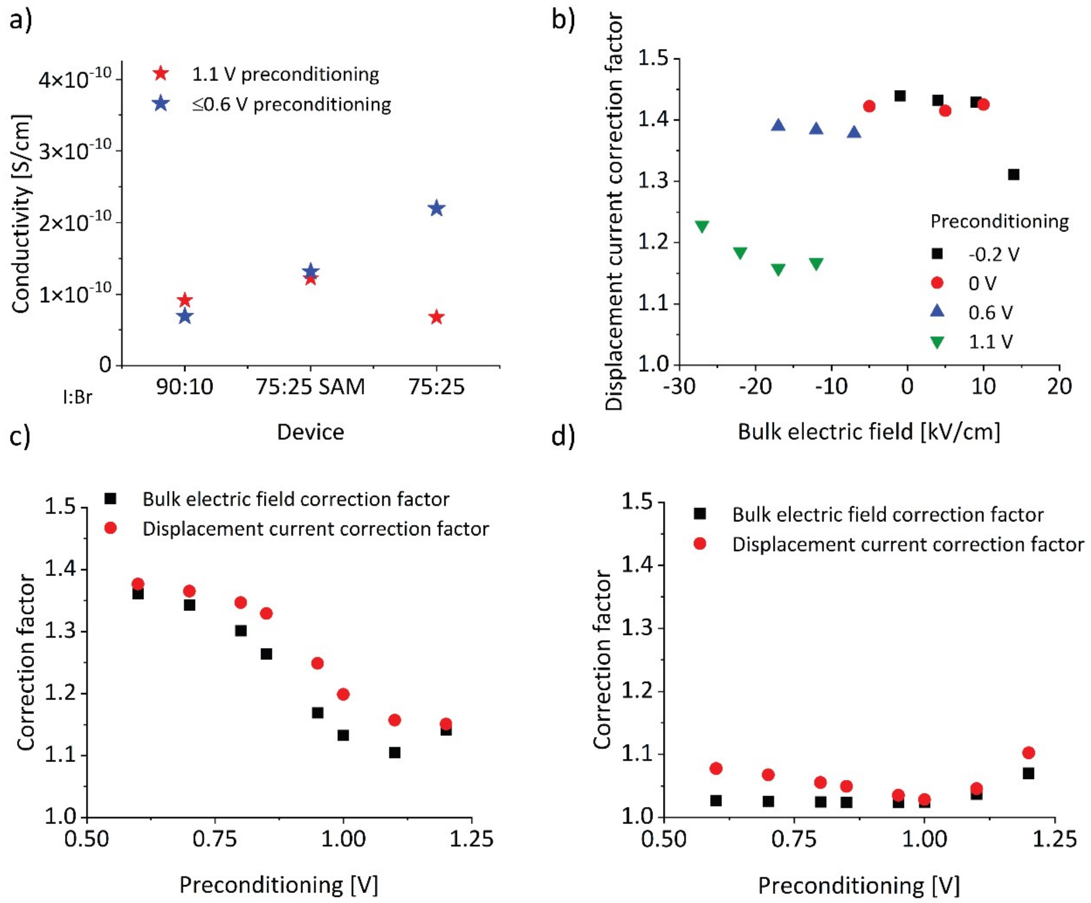

Fig. Note SN6 a) Extracted ion conductivities for preconditioning at 1.1 V and ≤ 0.6 V for the three investigated device structures. b) Ratio of total current density to cation drift current for the whole set of BACE measurements, indicated as correction factor. The correction is a result of the displacement current due to the electric field screening. c) Bulk electric field and displacement current correction factors for simulations with preconditioning from 1.3 V to 0.6 V and a subsequent switch to 0.25 V for the base model used for all previous simulation results, and in d) for a model with high TL doping.

References

1 Y.-H. Lin, N. Sakai, P. Da, J. Wu, H. C. Sansom, A. J. Ramadan, S. Mahesh, J. Liu, R. D. J. J. Oliver, J. Lim, L. Aspitarte, K. Sharma, P. K. Madhu, A. B. Morales-Vilches, P. K. Nayak, S. Bai, F. Gao, C. R. M. M. Grovenor, M. B. Johnston, J. G. Labram, J. R. Durrant, J. M. Ball, B. Wenger, B. Stannowski and H. J. Snaith, *Science*, 2020, **369**, 96–102.

2 M. Taddei, J. A. Smith, B. M. Gallant, S. Zhou, R. J. E. Westbrook, Y. Shi, J. Wang, J. N. Drysdale, D. P. McCarthy, S. Barlow, S. R. Marder, H. J. Snaith and D. S. Ginger, *ACS Energy Lett.*, 2022, **7**, 4265–4273.

3 P. Calado, I. Gelmetti, B. Hilton, M. Azzouzi, J. Nelson and P. R. F. Barnes, *J Comput Electron*, 2022, **21**, 960–991.

4 P. Calado, A. M. Telford, D. Bryant, X. Li, J. Nelson, B. C. O'Regan and P. R. F. Barnes, *Nature Communications*, 2016, **7**, 13831.
5 D. Moia, I. Gelmetti, P. Calado, W. Fisher, M. Stringer, O. Game, Y. Hu, P. Docampo, D. Lidzey, E. Palomares, J. Nelson and P. R. F. Barnes, *Energy & Environmental Science*, 2019, **12**, 1296–1308.
6 N. Phung, A. Al-Ashouri, S. Meloni, A. Mattoni, S. Albrecht, E. L. Unger, A. Merdasa and A. Abate, *Adv. Energy Mater.*, 2020, **10**, 1903735.
7 H. Wang, A. Guerrero, A. Bou, A. M. Al-Mayouf and J. Bisquert, *Energy and Environmental Science*, 2019, **12**, 2054–2079.
8 G. Y. Kim, A. Senocrate, D. Moia and J. Maier, *Adv. Funct. Mater.*, 2020, **30**, 2002426.
9 A. J. Knight and L. M. Herz, *Energy & Environmental Science*, 2020, **13**, 2024–2046.
10 F. Ebadi, B. Yang, Y. Kim, R. Mohammadpour, N. Taghavinia, A. Hagfeldt and W. Tress, *Journal of Materials Chemistry A*, 2021, **9**, 13967–13978.
11 A. J. Knight, J. Borchert, R. D. J. J. Oliver, J. B. Patel, P. G. Radaelli, H. J. Snaith, M. B. Johnston and L. M. Herz, *ACS Energy Letters*, 2021, **6**, 799–808.
12 A. J. Knight, J. B. Patel, H. J. Snaith, M. B. Johnston and L. M. Herz, *Advanced Energy Materials*, 2020, **10**, 1903488.
13 L. Krückemeier, B. Krogmeier, Z. Liu, U. Rau and T. Kirchartz, *Advanced Energy Materials*, 2021, **11**, 2003489.
14 S. Mahesh, J. M. Ball, R. D. J. J. Oliver, D. P. McMeekin, P. K. Nayak, M. B. Johnston and H. J. Snaith, *Energy & Environmental Science*, 2020, **13**, 258–267.
15 I. Karimata, K. Ohta, Y. Kobori and T. Tachikawa, *ACS Applied Materials & Interfaces*, 2018, **10**, 37057–37066.
16 X. Fu, K. J. Weber and T. P. White, *Journal of Applied Physics*, 2018, **124**, 073102.
17 M. Diethelm, M. Bauer, W.-H. Hu, C. Vael, S. Jenatsch, P. W. M. Blom, F. Nüesch and R. Hany, *Advanced Functional Materials*, 2022, **32**, 2106185.
18 P. Caprioglio, J. A. Smith, R. D. J. Oliver, A. Dasgupta, S. Choudhary, M. D. Farrar, A. J. Ramadan, Y.-H. Lin, M. G. Christoforo, J. M. Ball, J. Diekmann, J. Thiesbrummel, K.-A. Zaininger, X. Shen, M. B. Johnston, D. Neher, M. Stolterfoht and H. J. Snaith, *Nat Commun*, 2023, **14**, 932.
19 R. D. J. Oliver, P. Caprioglio, F. Peña-Camargo, L. R. V. Buizza, F. Zu, A. J. Ramadan, S. G. Motti, S. Mahesh, M. M. McCarthy, J. H. Warby, Y.-H. Lin, N. Koch, S. Albrecht, L. M. Herz, M. B. Johnston, D. Neher, M. Stolterfoht and H. J. Snaith, *Energy & Environmental Science*, 2022, **15**, 714–726.
20 J. Werner, G. Nogay, F. Sahli, T. C.-J. Yang, M. Bräuninger, G. Christmann, A. Walter, B. A. Kamino, P. Fiala, P. Löper, S. Nicolay, Q. Jeangros, B. Niesen and C. Ballif, *ACS Energy Lett.*, 2018, **3**, 742–747.
21 Y. Chen, S. G. Motti, R. D. J. Oliver, A. D. Wright, H. J. Snaith, M. B. Johnston, L. M. Herz and M. R. Filip, *J. Phys. Chem. Lett.*, 2022, **13**, 4184–4192.
22 D. Walter, J. Peng, K. Weber, K. R. Catchpole and T. P. White, *Energy Environ. Sci.*, 2022, **15**, 5202–5216.
23 J. Diekmann, F. Peña-Camargo, N. Tokmoldin, J. Thiesbrummel, J. Warby, E. Gutierrez-Partida, S. Shah, D. Neher and M. Stolterfoht, *J. Phys. Chem. Lett.*, 2023, **14**, 4200–4210.
24 D. A. Jacobs, H. Shen, F. Pfeffer, J. Peng, T. P. White, F. J. Beck and K. R. Catchpole, *Journal of Applied Physics*, 2018, **124**, 225702.
25 M. H. Futscher, J. M. Lee, L. McGovern, L. A. Muscarella, T. Wang, M. I. Haider, A. Fakharuddin, L. Schmidt-Mende and B. Ehrler, *Materials Horizons*, 2019, **6**, 1497–1503.

26 A. Zamouche, T. Heiser and A. Mesli, *Appl. Phys. Lett.*, 1995, **66**, 631–633.
27 I. Lyubomirsky, M. K. Rabinal and D. Cahen, *Journal of Applied Physics*, 1997, **81**, 6684–6691.
28 Y. Rakita, I. Lubomirsky and D. Cahen, *Materials Horizons*, 2019, **6**, 1297–1305.
29 N. Phung, R. Félix, D. Meggiolaro, A. Al-Ashouri, G. Sousa e Silva, C. Hartmann, J. Hidalgo, H. Köbler, E. Mosconi, B. Lai, R. Gunder, M. Li, K.-L. Wang, Z.-K. Wang, K. Nie, E. Handick, R. G. Wilks, J. A. Marquez, B. Rech, T. Unold, J.-P. Correa-Baena, S. Albrecht, F. De Angelis, M. Bär and A. Abate, *J. Am. Chem. Soc.*, 2020, **142**, 2364–2374.
30 M. H. Futscher and C. Deibel, *ACS Energy Letters*, 2022, **7**, 140–144.

Data

Fig. 1d

	Bias difference	Ion density
	[V]	[cm^{-3}]
Precond: -0.2V, Applied bias: 0.5V	0.7	1.69E+16
Precond: 0.6V, Applied bias: 0.5V	-0.1	-1.52E+14
Precond: 0.0V, Applied bias: 0.5V	0.5	2.43E+16
Precond: 1.1V, Applied bias: 0.5V	-0.6	-2.20E+16
Precond: -0.2V, Applied bias: 0.25V	0.45	5.56E+15
Precond: -0.2V, Applied bias: -0.25V	-0.05	-2.74E+14
Precond: 0.6V, Applied bias: 0.25V	-0.35	-4.54E+15
Precond: 0.6V, Applied bias: -0.25V	-0.85	-1.30E+16
Precond: 0.0V, Applied bias: 0.25V	0.25	4.34E+15
Precond: 0.0V, Applied bias: -0.25V	-0.25	-3.94E+15
Precond: 1.1V, Applied bias: 0.25V	-0.85	-3.22E+16
Precond: 1.1V, Applied bias: -0.25V	-1.35	-3.53E+16
Precond: -0.2V, Applied bias: 0.0V	0.2	2.04E+15
Precond: 0.6V, Applied bias: 0.0V	-0.6	-6.54E+15
Precond: 1.1V, Applied bias: 0.0V	-1.1	-2.69E+16

Fig 1e

Preconditioning	Applied bias	Bias difference	Bulk electric field	Current density	Current density
[V]	[V]	[V]	[kV/cm]	[µA/cm^2]	[µA/cm^2]
				Measurement	Simulation
-0.2	-0.25	-0.05	-1	-1.59E-01	-0.07471
-0.2	0	0.2	4	1.87E-01	0.2995
-0.2	0.25	0.45	9	5.82E-01	0.67437
-0.2	0.5	0.7	14	9.67E-01	1.1952
0	-0.25	-0.25	-5	-3.47E-01	-0.37045
0	0	0	0	-3.05E-02	1.62E-07
0	0.25	0.25	5	3.26E-01	0.37937

0	0.5	0.5	10	8.05E-01	0.75784
0.6	-0.25	-0.85	-17	-1.19E+00	-1.3394
0.6	0	-0.6	-12	-7.28E-01	-0.95225
0.6	0.25	-0.35	-7	-5.58E-01	-0.5599
0.6	0.5	-0.1	-2	-1.21E-01	-0.00815
1.1	-0.25	-1.35	-27	-3.05E+00	-3.0117
1.1	0	-1.1	-22	-2.12E+00	-2.5628
1.1	0.25	-0.85	-17	-2.09E+00	-2.0347
1.1	0.5	-0.6	-12	-1.53393	-1.4241

Fig. 3b

Dots

Device			90:10:00	75:25 SAM	75:25:00
Conductivity	[S/cm]	Precond: -0.2V, Applied bias: -0.25V	1.59E-10	1.65E-10	2.79E-10
Conductivity	[S/cm]	Precond: -0.2V, Applied bias: 0.0V	4.68E-11	9.08E-11	1.61E-10
Conductivity	[S/cm]	Precond: -0.2V, Applied bias: 0.25V	6.46E-11	1.27E-10	1.99E-10
Conductivity	[S/cm]	Precond: -0.2V, Applied bias: 0.5V	6.91E-11	1.73E-10	2.90E-10
Conductivity	[S/cm]	Precond: 0.0V, Applied bias: -0.25V	6.94E-11	1.21E-10	2.04E-10
Conductivity	[S/cm]	Precond: 0.0V, Applied bias: 0.0V	--	--	--
Conductivity	[S/cm]	Precond: 0.0V, Applied bias: 0.25V	6.52E-11	1.16E-10	1.95E-10
Conductivity	[S/cm]	Precond: 0.0V, Applied bias: 0.5V	8.05E-11	1.41E-10	2.46E-10
Conductivity	[S/cm]	Precond: 0.6V, Applied bias: -0.25V	7.03E-11	1.23E-10	1.96E-10
Conductivity	[S/cm]	Precond: 0.6V, Applied bias: 0.0V	6.07E-11	1.03E-10	1.71E-10
Conductivity	[S/cm]	Precond: 0.6V, Applied bias: 0.25V	7.97E-11	1.29E-10	2.27E-10
Conductivity	[S/cm]	Precond: 0.6V, Applied bias: 0.5V	6.07E-11	-1.16E-10	2.88E-10
Conductivity	[S/cm]	Precond: 1.1V, Applied bias: -0.25V	1.13E-10	1.83E-10	2.24E-10
Conductivity	[S/cm]	Precond: 1.1V, Applied bias: 0.0V	9.64E-11	1.29E-10	2.29E-10
Conductivity	[S/cm]	Precond: 1.1V, Applied bias: 0.25V	1.23E-10	1.63E-10	3.19E-10
Conductivity	[S/cm]	Precond: 1.1V, Applied bias: 0.5V	1.28E-10	2.52E-10	4.00E-10

Stars

Device	Slope BACE vs Ebulk plot	Conductivity
	[μA/cm^2]/[kV/cm]	[S/cm]
	<1.1 V preconditioning	<1.1 V preconditioning
90:10:00	0.06911	6.91E-11
75:25 SAM	0.13172	1.32E-10
75:25:00	0.2196	2.20E-10

Fig 4d

	Bias difference	Ion density
	[V]	[cm^{-3}]

Precond: -0.2V, Applied bias: 0.5V	0.7	6.00E+15
Precond: 0.6V, Applied bias: 0.5V	-0.1	2.20E+15
Precond: 0.0V, Applied bias: 0.5V	0.5	5.29E+15
Precond: 1.1V, Applied bias: 0.5V	-0.6	-5.79E+15
Precond: -0.2V, Applied bias: 0.25V	0.45	2.88E+15
Precond: -0.2V, Applied bias: -0.25V	-0.05	-2.94E+14
Precond: 0.6V, Applied bias: 0.25V	-0.35	-2.59E+15
Precond: 0.6V, Applied bias: -0.25V	-0.85	-5.74E+15
Precond: 0.0V, Applied bias: 0.25V	0.25	1.66E+15
Precond: 0.0V, Applied bias: -0.25V	-0.25	-1.52E+15
Precond: 1.1V, Applied bias: 0.25V	-0.85	-9.79E+15
Precond: 1.1V, Applied bias: -0.25V	-1.35	-1.25E+16
Precond: -0.2V, Applied bias: 0.0V	0.2	1.23E+15
Precond: 0.6V, Applied bias: 0.0V	-0.6	-4.25E+15
Precond: 1.1V, Applied bias: 0.0V	-1.1	-1.01E+16

Fig. 4e

	Bias difference	Ion density
	[V]	[cm^{-3}]
Precond: -0.2V, Applied bias: 0.5V	0.7	1.06E+16
Precond: 0.6V, Applied bias: 0.5V	-0.1	1.50E+16
Precond: 0.0V, Applied bias: 0.5V	0.5	-1.16E+16
Precond: 1.1V, Applied bias: 0.5V	-0.6	1.63E+16
Precond: -0.2V, Applied bias: 0.25V	0.45	3.44E+13
Precond: -0.2V, Applied bias: -0.25V	-0.05	-1.35E+12
Precond: 0.6V, Applied bias: 0.25V	-0.35	-6.03E+13
Precond: 0.6V, Applied bias: -0.25V	-0.85	-1.11E+14
Precond: 0.0V, Applied bias: 0.25V	0.25	2.88E+13
Precond: 0.0V, Applied bias: -0.25V	-0.25	-8.30E+12
Precond: 1.1V, Applied bias: 0.25V	-0.85	-5.28E+14
Precond: 1.1V, Applied bias: -0.25V	-1.35	-5.84E+14
Precond: -0.2V, Applied bias: 0.0V	0.2	7.10E+12
Precond: 0.6V, Applied bias: 0.0V	-0.6	-1.03E+14
Precond: 1.1V, Applied bias: 0.0V	-1.1	-5.80E+14

Fig. 5c

Preconditioning	Applied bias	Bulk electric field	Bulk electric field correction factor
[V]	[V]	[kV/cm]	
-0.2	0.25	9	1.39529
-0.2	0.5	14	1.4138
-0.2	-0.25	-1	1.39523
-0.2	0	4	1.39609

0	0.25	5	1.39219
0	0.5	10	1.39235
0	-0.25	-5	1.39383
0.6	0.25	-7	1.36107
0.6	-0.25	-17	1.36955
0.6	0	-12	1.36573
1.1	0.25	-17	1.10501
1.1	0.5	-12	1.10536
1.1	-0.25	-27	1.1205
1.1	0	-22	1.11055

Fig. 5d

Preconditioning	Bulk electric field correction factor base model	Bulk electric field correction factor High TL doping
[V]		
1.3	1.22569	1.13208
1.2	1.14167	1.07019
1.1	1.10501	1.03717
1	1.13262	1.02423
0.95	1.16909	1.02314
0.85	1.26391	1.02397
0.8	1.30148	1.02455
0.7	1.34312	1.02555
0.6	1.36107	1.02619
